# Identification of drought-responsive phenolic compounds and their biosynthetic regulation under drought stress in *Ligularia fischeri*

**DOI:** 10.3389/fpls.2023.1140509

**Published:** 2023-02-13

**Authors:** Yun Ji Park, Do Yeon Kwon, Song Yi Koo, To Quyen Truong, Sung-Chul Hong, Jaeyoung Choi, Jinyoung Moon, Sang Min Kim

**Affiliations:** ^1^ Smart Farm Research Center, KIST Gangneung Institute of Natural Products, Gangneung, Republic of Korea; ^2^ Euseed Inc, Daejeon, Republic of Korea; ^3^ Natural Product Informatics Center, KIST Gangneung Institute of Natural Products, Gangneung, Republic of Korea; ^4^ Department of Bio-medical Science & Technology, Korea Institute of Science and Technology (KIST) School, University of Science and Technology, Seoul, Republic of Korea

**Keywords:** *Ligularia fischeri*, drought stress, transcriptome, drought-responsive gene, phenylpropanoid biosynthesis, phenolic compounds, anthocyanin

## Abstract

*Ligularia fischeri*, a leafy edible plant found in damp shady regions, has been used as an herbal medicine and is also consumed as a horticultural crop. In this study, we investigated the physiological and transcriptomic changes, especially those involved in phenylpropanoid biosynthesis, induced by severe drought stress in *L. fischeri* plants. A distinguishing characteristic of *L. fischeri* is a color change from green to purple due to anthocyanin biosynthesis. We chromatographically isolated and identified two anthocyanins and two flavones upregulated by drought stress using liquid chromatography-mass spectrometry and nuclear magnetic resonance analyses in this plant for the first time. In contrast, all types of caffeoylquinic acids (CQAs) and flavonol contents were decreased under drought stress. Further, we performed RNA sequencing to examine the molecular changes in these phenolic compounds at the transcriptome level. In an overview of drought-inducible responses, we identified 2,105 hits for 516 distinct transcripts as drought-responsive genes. Moreover, differentially expressed genes (DEGs) associated with phenylpropanoid biosynthesis accounted for the greatest number of both up- and downregulated DEGs by Kyoto Encyclopedia of Genes and Genomes enrichment analysis. We identified 24 meaningful DEGs based on the regulation of phenylpropanoid biosynthetic genes. Potential drought-responsive genes included upregulated flavone synthase (*LfFNS*, TRINITY DN31661 c0 g1 i1) and anthocyanin 5-*O*-glucosyltransferase (*LfA5GT1*, TRINITY DN782 c0 g1 i1), which could contribute to the high levels of flavones and anthocyanins under drought stress in *L. fischeri*. In addition, the downregulated shikimate *O*-hydroxycinnamolytransferase (*LfHCT*, TRINITY DN31661 c0 g1 i1) and hydroxycinnamoyl-CoA quinate/shikimate transferase (*LfHQT4*, TRINITY DN15180 c0 g1 i1) genes led to a reduction in CQAs. Only one or two BLASTP hits for *LfHCT* were obtained for six different Asteraceae species. It is possible that the HCT gene plays a crucial role in CQAs biosynthesis in these species. These findings expand our knowledge of the response mechanisms to drought stress, particularly regarding the regulation of key phenylpropanoid biosynthetic genes in *L. fischeri*.

## 1 Introduction

*Ligulari fischeri*, also referred to as *Gomchi* in Korea, is a medicinal plant species in the family Asteraceae mn. This edible leafy plant is found in wet shady regions and is mainly distributed in China, Korea, Japan, Europe, and the far-east region of Russia (Xie et al., 2010). Notably, this plant is used as a cooking ingredient as well as a traditional remedy to treat various diseases in China and its leaves have been historically consumed as either fresh or salted fermented vegetables in Korea. In addition, *L. fischeri* leaf tea, which is brewed by blanching fresh leaves in hot water, has recently been considered a high-value nutritional food, and its primary ingredients in tea infusion have been investigated (Kim et al., 2010). Currently, *L. fischeri* can be found in the wild and is also massively cultivated in farms and greenhouses, with a continuous increase in its cultivation area over time to meet the commercial demand (Kim et al., 2012). Among herbal medicines, *L. fischeri* leaves have been used to treat hepatic failure, jaundice, rheumatoid arthritis, and scarlet fever (Park et al., 2016). In accordance with several *in vitro* and *in vivo* studies, *L. fischeri* has anti-hepatotoxic, anti-inflammatory, and anti-obesity properties (Kim et al., 2019). Moreover, the solvents and aqueous extracts of this plant show high radical-scavenging activities and are a key source of dietary antioxidants (Kim et al., 2010). Most biological activities can be attributed to the functional compounds found in plants. Species in the genus *Ligularia* have been reported to contain a wide variety of phytochemicals, including alkaloids, flavonoids, steroids, terpenoids, and other compounds associated with various biological activities (Yang et al., 2011). Previous studies have shown that the therapeutic effects of *L. fischeri* are determined by active compounds such as caffeoylquinic acid (CQA), sesquiterpenoids, phenolic compounds, terpenoids, monocyclosqualene, spiciformisins, and norsesquiterpene derivatives (Azam Ansari et al., 2019). Of these, CQA and most CQA derivatives have been identified as the predominant phenolic compounds in *L. fischeri* leaves (Shang et al., 2010).

Environmental constraints substantially influence plant growth and development, with effects ranging from mild to severe, based on the intensity and duration of stress and plant growth stage (Sourour et al., 2017). Several global regions are dealing with global warming-induced water shortages and droughts, which in turn result in reduced water uptake, restricting subsequent nutrient absorption and affecting crop growth, gene expression, distribution, productivity, and quality (Guo et al., 2018). Water scarcity impairs events involving cell mitosis, such as division, elongation, and turgor pressure, as well as critical enzymes responsible for nutrient assimilation, resulting in nutrient deficiency and reductions in height, leaf area, number, and plant biomass (Farooq et al., 2009). The most severe effect of drought is observed in photosynthesis, which is an essential process that provides energy for all metabolic activities in plants. Water shortages impair enzymes involved in carbon fixation, the electron transport chain, and photorespiration, and cause deterioration of the photosynthetic apparatus (Farooq et al., 2009). Moderate to severe water stress affects a variety of morpho-physiological properties, including chlorophyll fluorescence, water usage efficiency, dry matter output, water content, water potential, membrane ability, and pigment content stability (Sourour et al., 2017). Previous studies have revealed that the accumulation of organic and inorganic solutes is induced to maintain the actual intercellular water level and to promote carbon dioxide uptake *via* the stomata and water *via* roots. Moreover, the regulation of plant growth factors and phytohormones is also modified by drought stress. Plant cells have developed an antioxidant defense system because insufficient water supply is linearly associated with the increase in and accumulation of reactive oxygen species (Farooq et al., 2009). Therefore, plants need to develop a drought resistance system to overcome water constraints and survive under drought conditions.

Drought stress also causes a wide range of biochemical and physiological reactions that assist in the maintenance of water and ionic homeostasis and prevent plants from wilting and desiccation (Singh et al., 2017). For instance, glycolysis, hormone synthesis, photosynthesis, sugar synthesis, and the tricarboxylic acid cycle are involved in plant responses to drought stress (Guo et al., 2018). Drought-induced metabolites such as glycine-betaine, proline, and soluble sugars reduce the osmotic potential of cells and improve water infiltration in plants without disrupting normal metabolic processes. This reaction, referred to as osmotic adjustment, allows plants to sustain cell turgor for development and survival under stressful conditions (Takahashi et al., 2020). Plants activate an array of mechanisms for survival, especially the biosynthesis of secondary metabolites, including phenolics/flavonoids, when subjected to water deficits. Drought stress affects the accumulation of polyphenolic substances including anthocyanins and other flavonoids. For instance, flavonoids and anthocyanins in pea plants and graph berries are significantly increased by drought (Nogués et al., 1998; Castellarin et al., 2007). Additionally, stress-induced synthesis of antioxidants, such as flavonoids (e.g., flavonols and anthocyanins), enables plants to serve as free radical scavengers, mitigating oxidative and dehydration stress (Nakabayashi et al., 2014).

Several studies have investigated the effects of different environmental conditions, including weather, cultivation systems, and sunlight on the production of active compounds in *L. fischeri* (Kim et al., 2012; Rekha et al., 2015; Hao et al., 2018). We previously demonstrated that the major phenolic components, CQAs, were induced by sunlight exposure in *L. fischeri*. Because this plant is typically grown in moist and shady regions, several growth conditions can be altered to improve its functional constituents (Kim et al., 2012). In this study, we investigated the effect of severe drought stress in *L. fischeri* plants to evaluate its effect on polyphenolic composition and its induction of regulatory changes. We identified and characterized two flavonoids and two anthocyanins for the first time in this plant under drought stress. We compared changes in major phenolic compounds between the control and drought-treated plants. Furthermore, we performed RNA sequencing to determine differentially expressed genes and explore the drought-related network that is adapted to drought stress in *L. fischeri*.

## 2 Materials and methods

### 2.1 Reagents

Analytical grade and high-performance liquid chromatography (HPLC)-graded solvents were obtained from Daejung (Gyeonggi, Korea) and Fisher Scientific (Pittsburgh, PA, USA). Deuterated liquids for nuclear magnetic resonance (NMR) spectroscopy were obtained from the Cambridge Isotope Laboratory (Andover, MA, USA). Hyperoside, potassium persulfate, formic acid, and trifluoroacetic acid (TFA) were obtained from Sigma-Aldrich (St. Louis, MO, USA).

### 2.2 Plant materials

Three-year-old *L. fischeri* roots were grown under sunlight in Apr. 2019, in Gangneung, Korea. Plants were grown for 50 days under well-watered conditions. Subsequently, two groups of plants with two or three leaves each were established. The control group was maintained under the same conditions and the drought group was subjected to drought stress (with no additional water). Based on the data observed from Korea Meteorological Administration (http://data.kma.go.kr), the average temperature was 18.3°C (min 13.5°C – max 23.2°C) and the relative humidity was 54% during cultivation. After four weeks, the leaves were harvested from each group and dried under shade for one week for liquid chromatography-mass spectrometry (LC/MS) analysis and extraction for chromatography. Some leaves were stored at – 80°C for RNA extraction.

### 2.3 HPLC-MS and HPLC analysis

Dried leaves (200 mg) were extracted with 10 mL of methanol containing 0.1 N HCl for 12 h at room temperature. After filtration, the extract was analyzed using an Agilent LC-MS system (Agilent Technologies, Palo Alto, CA, USA), consisting of an analytical 1200 HPLC system with a Shiseido MG II C_18_ analytical column (250 × 4.6 mm i.d., 5 *μ*m particle size) and a 6120-quadrupole mass spectrometer with electrospray ionization (ESI). The mobile phase was made up of acetonitrile (A) and water (B), with 0.3% formic acid, respectively. The gradient was run as follows: 15% solvent A for 10 min, 15–40% solvent A for 18 min, 40–90% solvent A for 7 min, held at 90% solvent A for 5 min, and then returned to 15% solvent A for column equilibration. The separated components were detected at 330 nm for CQAs and flavonoids, and 520 nm for anthocyanins. Mass data were collected in full scan mode in positive ion mode from *m/z* 50 to 1000 under a 5 mL min^-1^ drying gas flow, 150°C vaporizing temperature, 60 psi nebulizing gas (N_2_) pressure, and 30°C drying gas temperature.

To determine and quantify phenolic compounds, we used the same HPLC conditions described above. An Agilent 1200 HPLC system equipped with a binary pump (G1312A), auto sampler (G1347B), PDA detector (G1315D), column oven (G1316A), and ChemStation, using an external standard method with a calibration curve, was used for HPLC analysis. In total, ten phenolic compounds, including four hydroxycinnamic acids, two flavonols, two flavones, and two anthocyanins, were used for calibration curve conduction in the range of 31.25 to 250 ppm. 2’’-Acetylhyperoside was quantified as a relatively equivalent value to hyperoside. All experiments were performed in triplicate.

### 2.4 Isolation of flavonoids and anthocyanins

*L. fischeri* leaves (300 g) were extracted thrice with methanol for 12 h at room temperature and then partitioned with *n*-hexane, dichloromethane, ethyl acetate, *n*-butanol, and water to obtain two unknown flavonoids. As indicated in a previous study, the ethyl acetate fractions were analyzed by HPLC (Kim et al., 2012). The mobile phase used for separation consisted of water and methanol. The methanol concentration was increased from 20 to 50% over 30 min at a flow rate of 10 mL min^-1^. Flavonoid compounds were detected at 330 nm. The two flavonoids were concentrated to yield 4.08 mg of Peak 3 and 5 mg of Peak 4.

For the two unknown anthocyanins, dried powder (150 g) of drought-treated leaves was extracted three times for 12 h each with methanol containing 0.1 N HCl. The extract was filtered and condensed using a vacuum evaporator. During purification, all solvents contained 0.1 N HCl to maintain the stability of anthocyanins. The crude extract (10 g) was dissolved in 100 mL of 0.1 N HCl and subjected to Amberlite XAD-7 HP (Supelco, Bellefonte, PA, USA) column chromatography (7 cm × 50 cm). The sample was eluted with aqueous methanol by gradually increasing the methanol concentration from 0 to 100%. Purple output fractions were collected and further purified by preparative HPLC as previously described (Kim et al., 2012). An acetonitrile gradient from 0 to 60% over 60 min was used to elute anthocyanins, and chromatogram peaks were observed at 520 nm. Peaks 9 and 10 were concentrated in the two anthocyanins, yielding 4 mg of peak 9 and 3 mg of peak **10**. In total, four compounds were successfully isolated and analyzed for structure determination and quantification.

### 2.5 NMR spectroscopy analysis

^1^H and ^13^C NMR data of the separated compounds were collected using a Varian NMR system operating at 500 MHz (Varian, Palo Alto, CA, USA).

#### 2.5.1 Luteolin-7-O-β-glucoside (peak 3)

This was a yellow powder; ^1^H NMR (500 MHz, DMSO-*d*_6_) *δ* 7.45 (dd, 1H, *J* = 2.5, 8.3 Hz, H-6′), 7.41 (d, 1H, *J* = 2.5 Hz, H-2′), 6.89 (d, 1H, *J* = 8.3 Hz, H-5′), 6.78 (d, 1H, *J* = 2.5 Hz, H-8), 6.76 (s, 1H, H-3), 6.44 (d, 1H, *J* = 2.5 Hz, H-6) (Luteolin moiety); *δ* 5.08 (d, 1H, *J* = 7.3 Hz, H-1′′), 3.15–3.71 (m, 5H, H-2′′–H-6′′) (Glucose moiety). ^13^C NMR (125 MHz, DMSO-*d*_6_) *δ* 182.4 (C-4), 164.9 (C-2), 163.4 (C-7), 161.6 (C-5), 157.4 (C-9), 150.4 (C-4′), 146.2 (C-3′), 121.8 (C-1′), 119.6 (C-6′), 116.4 (C-5′), 114.0 (C-2′), 105.8 (C-10), 103.6 (C-3), 100.0 (C-6), 95.1 (C-8) (Luteolin moiety); *δ* 100.2 (C-1′′), 77.6 (C-5′′), 76.8 (C-3′′), 73.5 (C-2′′), 69.9 (C-4′′), 61.0 (C-6′′) (Glucose moiety).

#### 2.5.2 Luteolin-7-O-β-glucuronide (peak 4)

This was a yellow powder; ^1^H NMR (500 MHz, DMSO-*d*_6_) *δ* 7.42 (d, 1H, *J* = 2.2 Hz, H-2′), 7.37 (dd, 1H, *J* = 2.2, 8.4 Hz, H-6′), 6.84 (d, 1H, *J* = 8.4 Hz, H-5′), 6.77 (d, 1H, *J* = 1.8 Hz, H-8), 6.68 (s, 1H, H-3), 6.39 (d, 1H, *J* = 1.8 Hz, H-6) (Luteolin moiety); *δ* 13.01 (s, 1H, -COOH), 5.08 (d, 1H, *J* = 7.3 Hz, H-1′′), 3.60 (d, 1H, *J* = 9.9 Hz, H-5′′), 3.26–3.40 (m, 3H, H-2′′–H4′′) (Glucuronide moiety). ^13^C NMR (125 MHz, DMSO-*d*_6_) *δ* 182.4 (C-4), 164.9 (C-2), 162.9 (C-7), 161.6 (C-5), 157.4 (C-9), 150.4 (C-4′), 146.2 (C-3′), 121.8 (C-1′), 119.6 (C-6′), 116.4 (C-5′), 114.0 (C-2′), 105.8 (C-10), 103.6 (C-3), 99.5 (C-6), 94.9 (C-8) (Luteolin moiety); *δ* 170.6 (C-6′′), 99.8 (C-1′′), 76.0 (C-3′′), 75.8 (C-5′′), 73.2 (C-2′′), 71.7 (C-4′′) (Glucuronide moiety).

#### 2.5.3 Cyanidin-3-O-β-glucoside (peak 9)

This was a purple powder; ^1^H NMR (500 MHz, CD_3_OD/TFA-*d*_1_ (9: 1, v/v)) *δ* 9.06 (s, 1H, H-4), 8.29 (dd, 1H, *J* = 2.3, 8.7 Hz, H-6′), 8.09 (d, 1H, *J* = 2.3 Hz, H-2′), 7.06 (d, 1H, *J* = 8.7 Hz, H-5′), 6.94 (d, 1H, *J* = 1.2 Hz, H-8), 6.70 (d, 1H, *J* = 2.0 Hz, H-6) (Cyanidin moiety); *δ* 5.33 (d, 1H, *J* = 7.8 Hz, H-1′′), 3.40–4.00 (m, 5H, H-2′′–H-6′′) (Glucose moiety). ^13^C NMR (125 MHz, CD_3_OD/TFA-*d*_1_ (9: 1, v/v)) *δ* 171.0 (C-7), 165.2 (C-2), 160.2(C-5), 158.7 (C-9), 156.7 (C-4′), 148.3 (C-3′), 146.6 (C-3), 137.9 (C-4), 129.1 (C-6′), 122.2 (C-1′), 119.3 (C-2′), 118.2 (C-5′), 114.2 (C-10), 104.6 (C-6), 96.0(C-8) (Cyanidin moiety); *δ* 104.2 (C-1′′), 79.7 (C-5′′), 79.0 (C-3′′), 75.6 (C-2′′), 71.9 (C-4′′), 63.2 (C-6′′) (Glucose moiety).

#### 2.5.4 Cyanidin-3-O-β-(6′′-malonylglucoside) (peak 10)

This was a purple powder; ^1^H NMR (500 MHz, CD_3_OD/TFA-*d*_1_ (9: 1, v/v)) *δ* 8.79 (s, 1H, H-4), 8.21 (dd, 1H, *J* = 2.4, 8.8 Hz, H-6′), 7.97 (d, 1H, *J* = 2.4 Hz, H-2′), 7.01 (d, 1H, *J* = 8.8 Hz, H-5′), 6.87 (d, 1H, *J* = 1.2 Hz, H-8), 6.69 (d, 1H, *J* = 2.0 Hz, H-6) (Cyanidin moiety); *δ* 5.37 (d, 1H, *J* = 7.8 Hz, H-1′′), 4.44 (m, 2H, H-6′′), 3.80 (m, 1H, H-5′′), 3.51 (t, 1H, H-2′′), 3.40 (t, 1H, H-3′′), 3.35 (d, 2H, *J* = 2.2 Hz, H-2′′′), 3.23 (t, 1H, H-4′′) (Malonylglucose moiety). ^13^C NMR (125 MHz, CD_3_OD/TFA-*d*_1_ (9: 1, v/v)) *δ* 168.6 (C-7), 162.0 (C-2), 157.7 (C-5), 156.4 (C-9), 154.6 (C-4′), 146.3 (C-3′), 144.4 (C-3), 134.7 (C-4), 127.2 (C-6′), 119.8 (C-1′), 117.6 (C-2′), 117.0 (C-5′), 112.0 (C-10), 102.6 (C-6), 94.4 (C-8) (Cyanidin moiety); *δ* 168.2 (C-3′′′), 167.3(C-1′′′), 101.9 (C-1′′), 76.3 (C-3′′), 74.5 (C-5′′), 73.1 (C-2′′), 70.1 (C-4′′), 64.6 (C-6′′), 41.4 (C-2′′′) (Malonylglucose moiety).

### 2.6 RNA extraction and RNA sequencing

Frozen leaf material (100 mg) was ground in liquid nitrogen, and RNA was extracted using Trizol (Invitrogen, Carlsbad, CA, USA) and the Qiagen RNeasy Plant Mini Kit (Qiagen, Hilden, Germany) according to the manufacturers’ instructions. RNase-free DNase I was used to remove genomic DNA. A NanoDrop ND-1000 spectrometer was used to measure the quality and quantity of RNA. RNA was run on an Agilent 2100 Bioanalyzer to determine RNA integrity. RNA integrity number (RIN) values were > 8.

Six RNA-Seq libraries were created from three biological replicates using a TruSeq RNA Library Prep Kit (Illumina Inc. San Diego, CA, USA) using SEEDERS (Daejeon, Republic of Korea) and processed on an Illumina HiSeq X system. In the pre-processing steps, adapter sequences were removed using Cutadapt and then trimmed using the DynamicTrim and LengthSort of the SolexaQA package (Cox et al., 2010; Martin, 2011). RNA-Seq data were deposited in the NCBI Sequencing Read Archive public database with Accession No number PRJNA626533, consisting of six libraries (Accession No. SRR11585851 to SRR11585856).

### 2.7 *De novo* transcriptome assembly and read mapping

The cleaned reads were assembled according to the manufacturer’s protocol using the Trinity program (v2.8.6) (https://github.com/trinityrnaseq/trinityrnaseq/wiki) (Grabherr et al., 2011). In addition, read counts were calculated by mapping clean reads, which were obtained as the total mapping reads of each transcript, using Bowtie2 (v2.1.0) software (Langmead and Salzberg, 2012). Mismatches were ≤ 2 bp. After read mapping, the number of cleaned reads was normalized using the DESeq library in R to avoid bias due to differences in coverage depth between the genes (Anders and Huber, 2010).

### 2.8 Functional annotation and differentially expressed genes (DEGs) analysis

To characterize the functions of the genes, annotation was conducted using amino acid sequences of Viridiplantae DB from NCBI NR and BLASTX (e-value ≤ 1e-10). InterProscan fulfilled the default criteria using the tools offered by EMBL. To determine the DEGs, fold change (FC) in gene expression and a bionomical test (adjusted *p*-value ≤ 0.01) were used. In this study, we selected DEGs with 2-fold or greater changes (log_2_FC ≥ 1 for upregulation and log_2_FC ≤ -1 for downregulation). All DEGs were subjected to gene ontology (GO) and Kyoto Encyclopedia of Genes and Genomes (KEGG) enrichment analyses using in-house scripts.

### 2.9 Taxonomic analysis

The datasets of translated representative transcripts were processed by aligning the contigs against the NCBI NR protein database, which was provided on Oct. 18, 2022, with the default parameter (E-value < 1e-30). In addition, the metagenomic community was visualized using Krona (version 2.8.1) (Ondov et al., 2011). Further, the major transcripts related to phenylpropanoid biosynthesis were translated and compared with the proteome sequences of six species of Asteraceae. The same E-value threshold (1e-30) was applied to remove spurious hits.

### 2.10 Statistical analysis

Data are expressed as mean ± standard deviation of three biological replicates. The two groups were compared using unpaired Student’s t-test. Differences were considered statistically significant at *p* < 0.05.

## 3 Results

### 3.1 Identification of phenolic compounds and drought stress-induced effects on metabolic events

**Figure 1:**
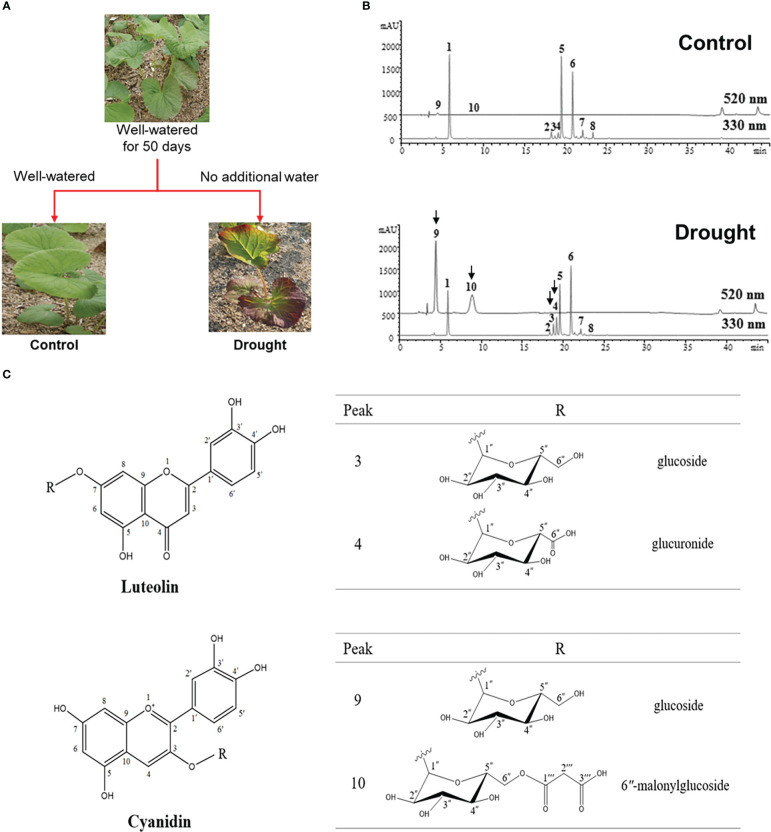
**(A)** Experimental scheme of phenotypes of *L. fischeri* under drought stress **(B)** Representative HPLC chromatograms of leaf extracts from control and drought treatments, monitored at 330 nm and 520 nm. Peak 1, 5-*O*-Caffeolyquinic acid; 2, Hyperoside; 3, Luteolin-7-*O*-β-glucoside; 4, Luteolin-7-*O*-*β*-glucuronide; 5, 3,4-di-*O*-Caffeoylquinic acid; 6, 3,5-di-*O*-Caffeoylquinic acid; 7, 4,5-di-*O*-Caffeoylquinic acid; 8, 2″-Acetylhyperoside; 9, Cyanidin-3-*O*-*β*-glucoside; and 10, Cyanidin-3-*O*-*β*-(6″-malonylglucoside). **(C)** Chemical structures of flavones and anthocyanins newly isolated from *L. fischeri* leaves.

We investigated the response of *L. fischeri* plants to drought by not watering them for four weeks. As shown in **Figure 1A**, the color change (from green to purple) was the most apparent feature of the plant. We determined the phytochemical changes in *L. fischeri* leaves under drought stress by analyzing leaf extracts using HPLC-MS analysis (**Figure 1B**). Preliminary identification of constituents in the leaf extract was conducted based on the UV-Vis absorption chromatogram (λ_max_ value) and fragmentation pattern using ESI-MS in the full scan mode (**Table 1**). Eight peaks (peaks **1**–**8**) and two peaks (peaks **9** and **10**) were observed at 330 and 520 nm, respectively. These compounds were identified by comparing their spectroscopic data with those of previous *L. fischeri* studies (Kim et al., 2012). According to the reported MS data, peaks **1**, **5**, **6**, and **7** were classified as derivatives of hydroxycinnamic acid (λ_max_ 330 nm) and identified as CQA, 3,4-di-*O*-caffeoylquinic acid, 3,5-di-*O*-caffeoylquinic acid, and 4,5-di-*O*-caffeoylquinic acid, respectively. Peaks **2** and **8** shared the same carbon skeletons of typical flavonoids, with molecular ions at *m/z* 464 and 507 in the positive mode, respectively. These two peaks were determined to be hyperoside (λ_max_ 256 and 357 nm) and 2″-acetylhyperoside (λ_max_ 256 and 353 nm), respectively (Piao et al., 2009; Park et al., 2016).

**Table 1:** UV/Vis and ESI/MS data of phenolic compounds in *L. fischeri* leaves.

Peak	Compound	UV/Vis (λmax, nm)	[M+H]^+^ (*m/z*)
1	5-*O*-Caffeoylquinic acid	326, 298	355
2	Hyperoside	357, 256	465
3	Luteolin-7-*O*-*β*-glucoside	348, 260	449
4	Luteolin-7-*O*-*β*-glucuronide	348, 260	463
5	3,4-di-*O*-Caffeoylquinic acid	330, 232	517
6	3,5-di-*O*-Caffeoylquinic acid	330, 246	517
7	4,5-di-*O*-Caffeoylquinic acid	330, 246	517
8	2″-Acetylhyperoside	353, 256	507
9	Cyanidin-3-*O*-*β*-glucoside	517, 282	450
10	Cyanidin-3-*O-β*-(6″-malonylglucoside)	517, 282	536

Peaks 3 and 4 were significantly induced in drought-treated samples, and their MS data have not been reported for this plant until now. Therefore, these compounds were isolated using chromatographic methods and subjected to NMR analysis to determine their chemical structures. Based on the ^1^H and ^13^C NMR spectral data, peaks 3 and 4 shared the same carbon skeleton of luteolin as the aglycon moiety of the flavonoids (**Figure 1C**) (Lee et al., 2011; Guvenalp et al., 2015). According to the ^13^C NMR data, peak **3** contains six extra carbons, except for the luteolin moiety. Its chemical shifts of ^1^H and ^13^C NMR indicated a glucosyl moiety (*δ*_H_ 5.08 and 3.15−3.71/*δ*_C_ 100.2, 77.6, 76.8, 73.5, 69.9, and 61.0) attached to the C-7 position. The glycone part was confirmed by ESI-MS in the positive mode with molecular ions at *m/z* 449 [M + H]^+^. Meanwhile, the ^13^C NMR data of peak 4 were slightly different from those of peak **3** in that there were seven extra carbons. The chemical shifts of ^1^H and ^13^C NMR suggested that a glucuronosyl moiety (*δ*_H_ 5.08, 3.26−3.60, and 13.01/*δ*_C_ 170.6, 99.8, 76.0, 75.8, 73.2, and 71.7) was attached to the same position of peak 3 with molecular ions at m/z 463 [M + H]^+^. Thus, peaks 3 and 4 were identified as luteolin-7-*O*-glucoside and luteolin-7-*O*-glucuronide, respectively (Fecka and Turek, 2008).

As shown in **Figure 1C**, peaks 9 and 10 corresponding to two anthocyanins were purified and their chemical structures were characterized using the same procedures described above. In the positive ion mode, the molecular formulae of these compounds were confirmed by ESI-MS with molecular ions at *m/z* 450 and 536 [M + H]^+^, respectively. With the analysis of ^1^H and ^13^C NMR spectral data, the characteristic signals of cyanidin aglycon at the C-4 position were observed with chemical shifts of *δ*_C_ 137.9 and *δ*_H_ 9.06 for peak 9, and *δ*_C_ 134.7 and *δ*_H_ 8.79 for peak 10. In the glycone part, peak 9 had the same glucosyl group attached to a different position (C-3) than that of peak 3 (C-7). Signals of one anomeric proton and the other methines were confirmed as a sugar moiety with the range of *δ*_H_ 3.40–5.33 for peak 9. In contrast, the remaining carbons (*δ*_C_ 168.2, 167.3, and 41.4) of peak 10 indicated a substitution with 6′′-malonylglucoside at the C-3 position. Moreover, the molecular ions detected at *m/z* 450 and 536 [M + H]^+^ suggest that peaks 9 and 10 were cyanidin-3-*O*-glucoside and cyanidin-3-*O*-(6′′-malonylglucoside), respectively (Narayan and Venkataraman, 2000).

The coupling constants of anomeric protons of each peak (*δ*_H_ 5.08, *J* = 7.3 Hz for peaks 3 and **4/** *δ*_H_ 5.33, *J* = 7.8 Hz for peak 9/*δ*_H_ 5.37, *J* = 7.8 Hz for peak 10) indicated that all of the glycone units were *β*-forms. Peaks 3, 4, 9, and 10 were identified as luteolin-7-*O*-*β*-glucoside, luteolin-7-*O*-*β*-glucuronide, cyanidin-3-*O*-*β*-glucoside, and cyanidin-3-*O*-*β*-(6′′-malonylglucoside), respectively, and were first isolated from *L. fischeri*.

According to our previous work, the major phenolic compounds in *L. fischeri* are hydroxycinnamic acid derivatives such as 5-*O*-caffeoylquinic acid and di-caffeoylquinic acids. These compounds accounted up to 9% of the phenolic compounds in the dried leaves of *L. fischeri* (Kim et al., 2012). In the present study, the major hydroxycinnamic acid derivatives in *L. fischeri* were downregulated after drought treatment (**Table 2**). Moreover, the content of two flavonols, hyperoside and 2″-acetylhyperoside, was decreased in drought-treated *L. fischeri* leaves. However, the newly identified flavones, luteolin-7-*O*-*β*-glucoside (peak 3) and luteolin-7-*O*-*β*-glucurunide (peak 4), were increased 2-fold and 3.34-fold, respectively, under drought stress. The content of two anthocyanins, including cyanidin-3-*O*-*β*-glucoside (peak 9) and cyanidin-3-*O*-*β*-(6″-malonylglucoside) (peak 10) was also significantly upregulated, corresponding to the color change. These appear to be the main changes brought on by drought stress in this plant.

**Table 2:** Contents of phenolic compounds in *L. fischeri* under drought stress (mg/g dry weight).

Subgroup	Compound	Control	Drought
Hydroxycinna-mic acid	5-*O*-Caffeoylquinic acid	16.46 ± 0.87	7.26 ± 0.25^**^
	3,4-di-*O*-Caffeoylquinic acid	13.83 ± 0.49	7.70 ± 0.21^**^
	3,5-di-*O*-Caffeoylquinic acid	18.31 ± 0.10	14.09 ± 0.32^**^
	4,5-di-*O*-Caffeoylquinic acid	7.16 ± 0.34	3.05 ± 0.10^**^
Flavonol	Hyperoside	4.38 ± 0.15	2.33 ± 0.08^**^
	2″-Acetylhyperoside	3.72 ± 0.16	0.32 ± 0.03^**^
Flavone	Luteolin-7-*O*-*β*-glucoside	1.03 ± 0.03	2.06 ± 0.04^**^
	Luteolin-7-*O*-*β*-glucuronide	2.86 ± 0.22	9.54 ± 0.34^**^
Anthocyanin	Cyanidin-3-*O*-*β*-glucoside	0.09 ± 0.01	2.79 ± 0.16^*^
	Cyanidin-3-*O-β*-(6″-malonylglucoside)	0.07 ± 0.02	1.82 ± 0.08^**^

Each value represents the mean ± SD of triplicate experiments; the control and drought groups were compared using an unpaired Student’s t-test (* *p* < 0.05; ** *p* < 0.01).

### 3.2 Sequence generation and transcript assembly

For transcriptome analysis, we generated cDNA libraries of the control and drought groups using Illumina RNA Sequencing. *L. fischeri* leaves were sequenced in triplicate, and the raw reads are available online in the SRA database at NCBI with the accession number PRJNA626533. Clean reads, ranging from 13.55 to 17.33 million, were obtained by pre-processing with an average of 93.18 to 94.24% read mapping efficiency. The number of reads generated for each sample is shown in **Supplementary Table 1**. Clean reads from the samples were used to generate the assembled transcriptome of *L. fischeri* using the Trinity program. The total assembled transcripts consisted of 74,873 transcripts, with an N50 value of 1,583 bp (**Supplementary Table 2**). Of these, 35,084 transcripts are indicated as representative, with an N50 value of 1,424 bp. The assembled transcripts ranged in length from 500 kb to > 14 kb. Prior to DEG analysis, a total of 4,122 DEGs were identified as being greater than the thresholds (*p*-value ≤ 0.001, log_2_FC ≥ 1 or ≤ -1) after read normalization. Overall, differential gene expression in the drought-treated and control groups revealed 1,896 upregulated and 2,792 downregulated transcripts. Of these, 1,514 up-regulated and 2,608 down-regulated genes were annotated using the NR public database (**Supplementary Figure 1A**). All DEGs were extracted from the expression matrix, and the results are shown in a volcano plot (**Supplementary Figure 1B**).

### 3.3 Functional enrichment analysis of DEGs

**Figure 2:**
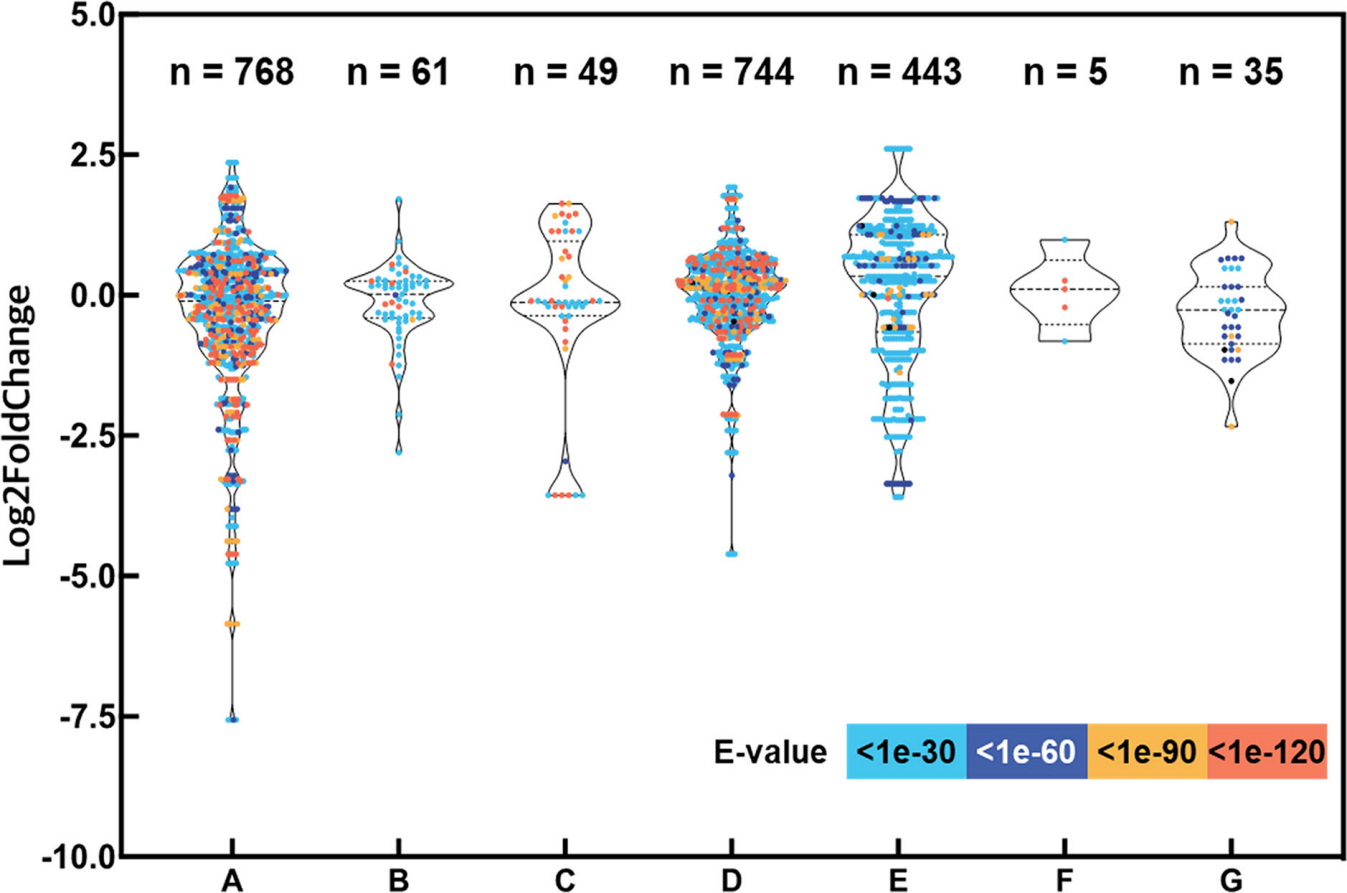
Expression levels of transcripts aligned with the drought-responsive genes by the seven categories and evaluation of the homology between the protein sequences encoding drought-responsive genes. The y-axis shows log2 fold change of each transcript and the x-axis indicates the gene categories shown in **Supplementary Table S3**: **(A)** ABA signaling, **(B)** Phosphorylation, **(C)** Osmotic stress sensing, **(D)** MAPK and Calcium signaling, **(E)** Transcription factors regulating gene expression during drought stress, **(F)** Epigenetic regulation, and **(G)** Drought stress-inducible genes encoding functional proteins. The number of hits (E-value < 1e-30) are shown at the top of the figure. The dots are displayed in different colors based on E-values from the homology searches.

Based on recent advances in drought stress signaling in plants, we selected numerous putative drought-responsive genes and classified them into several regulatory processes, such as abscisic acid (ABA) signaling, phosphorylation, osmotic stress sensing, MAPK and calcium signaling, transcription factors regulating gene expression during drought stress, epigenetic regulation, and drought stress-inducible genes encoding functional proteins (**Supplementary Table S3**) (Takahashi et al., 2018). A total of 75 protein sequences encoded by the drought-responsive genes of Arabidopsis were searched against representative transcripts of *L. fischeri* using BLASTP (E-value < 1e-30). As a result, 2,105 hits for 516 distinct transcripts were identified, and their distribution of expression levels showed intricate patterns, ranging across up- and downregulation under drought stress in *L. fischeri* (**Figure 2**).

**Figure 3:**
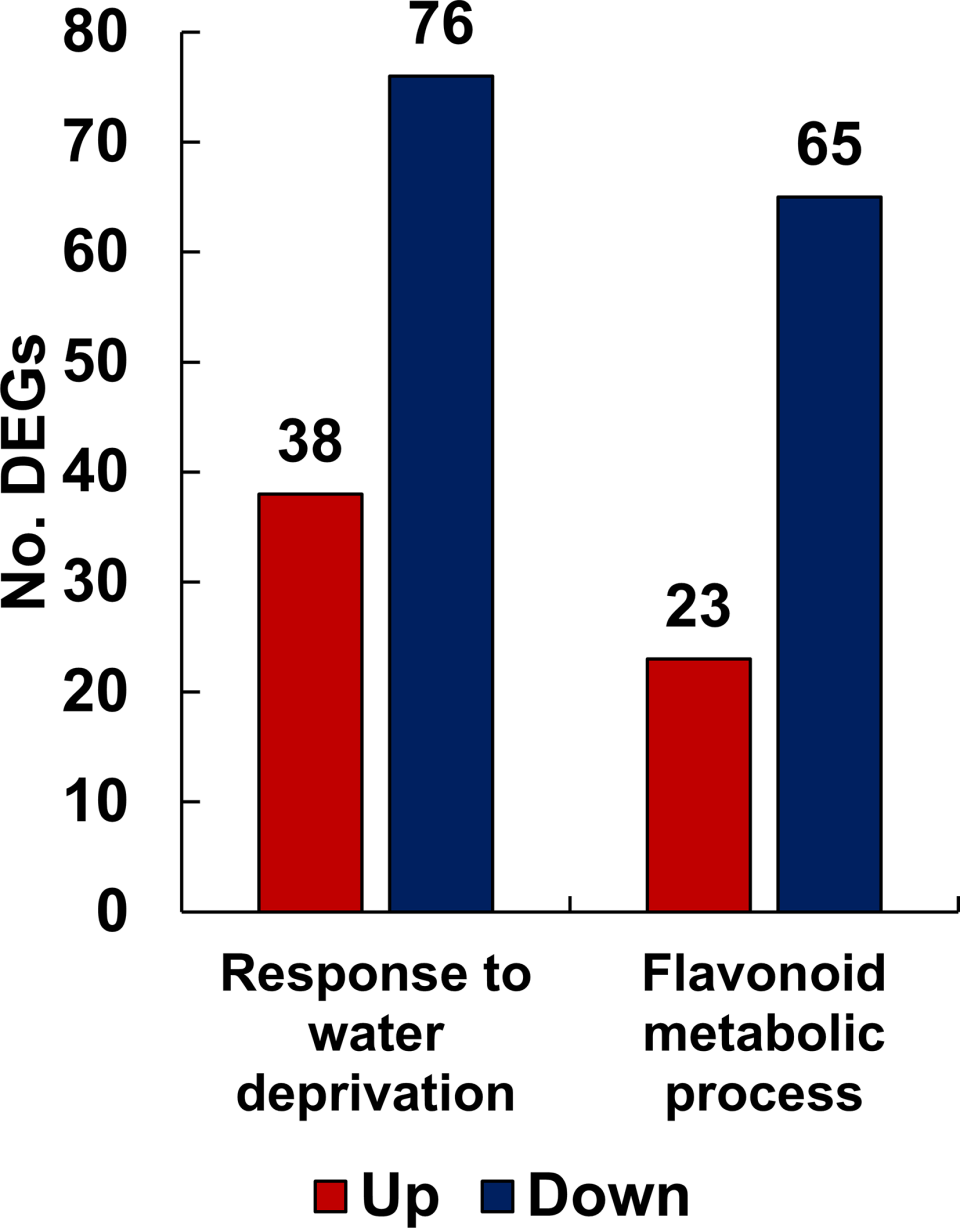
The number of up- and down-regulated DEGs related to response to water deprivation and flavonoid metabolic process functionally categorized by GO analysis from *L. fischeri* under drought stress. The x-axis represents the number of DEGs and the y-axis indicates each subcategory’s information. In bar graphs, red and blue colors are shown as up- and down-regulated DEGs, respectively.

To understand the function of the genes affected by drought stress, DEGs were classified into functional groups using GO analysis based on a corrected *p*-value of 0.05. Among the various GO classifications, we isolated the specific subcategories “response to water deprivation” and “flavonoid metabolic process” from the main functional category, biological processes. The term “response to water deprivation” includes any process that induces a change in the state or activity of a cell or organism through enzyme production, gene expression, movement, and secretion, as a result of a water scarcity stimulus. The flavonoid biosynthetic pathway is enhanced or suppressed by drought stress in *L. fischeri*. As shown in **Figure 3**, the number of downregulated genes outnumbered the number of upregulated genes in both the groups. Specifically, the “response to water deprivation” gene set included 38 upregulated and 76 downregulated genes. Moreover, the “flavonoid metabolic process” gene set contained 23 upregulated and 65 downregulated genes, further confirming the metabolic changes observed in *L. fischeri* under drought stress. The gene information for the DEGs associated with these subcategories is described in **Supplementary Table S4**.

To better explain the metabolic functions of the DEGs in *L. fischeri* leaves under drought stress, KEGG enrichment analysis was performed using the KEGG database and BLASTX, where the e-value of the filter standard was ≤ 1e-10. Based on the number of assigned DEGs, an enrichment analysis of KEGG terms related to DEGs was performed to further understand the potential metabolic roles in secondary metabolism in *L. fischeri* under drought stress. DEGs responsible for phenylpropanoid biosynthesis were most abundant in both up- (21) and downregulated (59) DEGs (**Table 3**). In addition, many DEGs were annotated for flavonoid biosynthesis, including 33 down-regulated and 12 up-regulated DEGs. These results are consistent with our findings that the contents of hydroxycinnamic acids and some flavonoids decrease under drought stress.

**Table 3:** KEGG pathway annotation and number of DEGs in biosynthesis of other secondary metabolites.

KEGG pathway item	Up	Down
Caffeine metabolism	1	0
Monobactam biosynthesis	2	4
Phenylpropanoid biosynthesis	21	59
Flavonoid biosynthesis	12	33
Anthocyanin biosynthesis	0	1
Isoflavonoid biosynthesis	2	4
Flavone and flavonol biosynthesis	0	5
Stilbenoid, diarylheptanoid and gingerol biosynthesis	9	18
Isoquinoline alkaloid biosynthesis	1	3
Tropane, piperidine and pyridine alkaloid biosynthesis	1	3
Glucosinolate biosynthesis	3	4
Biosynthesis of various secondary metabolites - part 2	0	2

### 3.4 DEGs in phenylpropanoid biosynthesis

As described above, various functional metabolites were significantly altered due to drought stress in *L. fischeri*. We observed that the contents of hydroxycinnamic acids and flavonols were downregulated, whereas the newly identified flavones and anthocyanins were significantly increased under drought stress. The principal cause of drought-induced phenolic compound production is regulation of the phenylpropanoid biosynthetic pathway. Drought modulates numerous essential genes responsible for phenylpropanoid biosynthesis and drought-induced changes in gene expression. Therefore, we isolated DEGs-related to phenylpropanoid biosynthesis based on the BLAST tool (TBLASTN) with an e-value cutoff of 1 × e^-10^ and identity ≥ 40%, and examined the distinct changes in *L. fischeri* subjected to drought stress. As shown in **Table 4**, we identified 24 DEGs encoding multiple enzymes associated with phenylpropanoid biosynthesis. This included nearly all of the enzymes required in the general pathway, including phenylalanine ammonia-lyase (*PAL*), cinnamate-4-hydroxylase (*C4H*), 4-coumarate:CoA ligase 1 (*4CL1*), chlacone synthase (*CHS*), chalcone isomerase (*CHI*), flavone synthase (*FNS*), flavanone 3-hydroxylase (*F3H*), flavonoid 3’ -hydroxylase (*F3’H*), anthocyanin 5-*O*-glucosyltransferase (*A5GT*), 4-coumarate 3-hydroxylase (*C3H*), shikimate *O*-hydroxycinnamoyltransferase (*HCT*), and hydroxycinnamoyl-CoA quinate/shikimate transferase (*HQT*).

**Table 4:** List of genes related to phenylpropanoid biosynthesis in *L. fischeri*.

Category	Gene ID	Symbol	Uniprot ID	Identity (%)	FC*
Upstream	TRINITY_DN299_c0_g1_i1	*LfPAL1*	AT2G37040	83.10	-1.89
	TRINITY_DN299_c0_g2_i1	*LfPAL2*		82.88	-2.00
	TRINITY_DN22101_c0_g1_i2	*LfC4H*	AT2G30490	40.00	0.74
	TRINITY_DN399_c0_g2_i1	*Lf4CL1*	AT1G51680	70.42	-1.85
Flavone	TRINITY_DN612_c0_g1_i1	*LfCHS*	AT5G13930	85.71	0.44
	TRINITY_DN772_c0_g2_i3	*LfCHI*	AT3G55120	59.36	-3.80
	TRINITY_DN59627_c0_g1_i1	*LfFNS*	AT5G07990	43.24	1.43
Flavonol	TRINITY_DN26907_c0_g1_i1	*LfF3H1*	AT3G51240	90.91	-1.24
	TRINITY_DN505_c0_g1_i1	*LfF3H2*		79.30	-1.40
	TRINITY_DN505_c0_g2_i1	*LfF3H3*		77.03	-1.44
	TRINITY_DN3519_c0_g1_i1	*LfF3’H1*	AT5G07990	70.11	-1.22
	TRINITY_DN6034_c0_g2_i4	*LfF3’H2*		46.93	-1.29
	TRINITY_DN1861_c0_g2_i2	*LfF3’H3*		42.29	-2.13
	TRINITY_DN81_c0_g1_i1	*LfF3’H4*		41.20	-2.16
Anthocyanin	TRINITY_DN782_c0_g1_i1	*LfA5GT1*	AT4G14090	42.64	1.36
	TRINITY_DN12729_c0_g1_i1	*LfA5GT2*		40.18	-4.03
Hydroxycinnamic acid	TRINITY_DN2611_c1_g1_i5	*LfC3H1*	AT2G40890	46.55	1.30
	TRINITY_DN6034_c0_g2_i4	*LfC3H2*		40.57	-1.29
	TRINITY_DN11373_c0_g3_i1	*LfC3H3*		43.56	-3.88
	TRINITY_DN31661_c0_g1_i1	*LfHCT*	AT5G48930	79.62	-3.99
	TRINITY_DN5048_c0_g1_i1	*LfHQT1*	AT5G48930	57.24	1.07
	TRINITY_DN12670_c0_g1_i1	*LfHQT2*		59.18	1.29
	TRINITY_DN430_c0_g1_i6	*LfHQT3*		56.16	1.07
	TRINITY_DN15180_c0_g1_i1	*LfHQT4*		67.19	-2.35

*Log_2_FoldChange. PAL, phenylalanine ammonia-lyase; C4H, cinnamate-4-hydroxylase; 4CL1, 4-coumarate:CoA ligase 1, CHS, chlacone synthase; CHI, chalcone isomerase; FNS, flavone synthase; F3H, flavanone 3-hydroxylase; F3’H, flavonoid 3’-hydroxylase; A5GT, anthocyanin 5-O-glucosyltransferase; C3H, 4-coumarate 3-hydroxylase; HCT, shikimate O-hydroxycinnamoyltransferase; HQT, hydroxycinnamoyl-CoA quinate/shikimate transferase.

**Figure 4:**
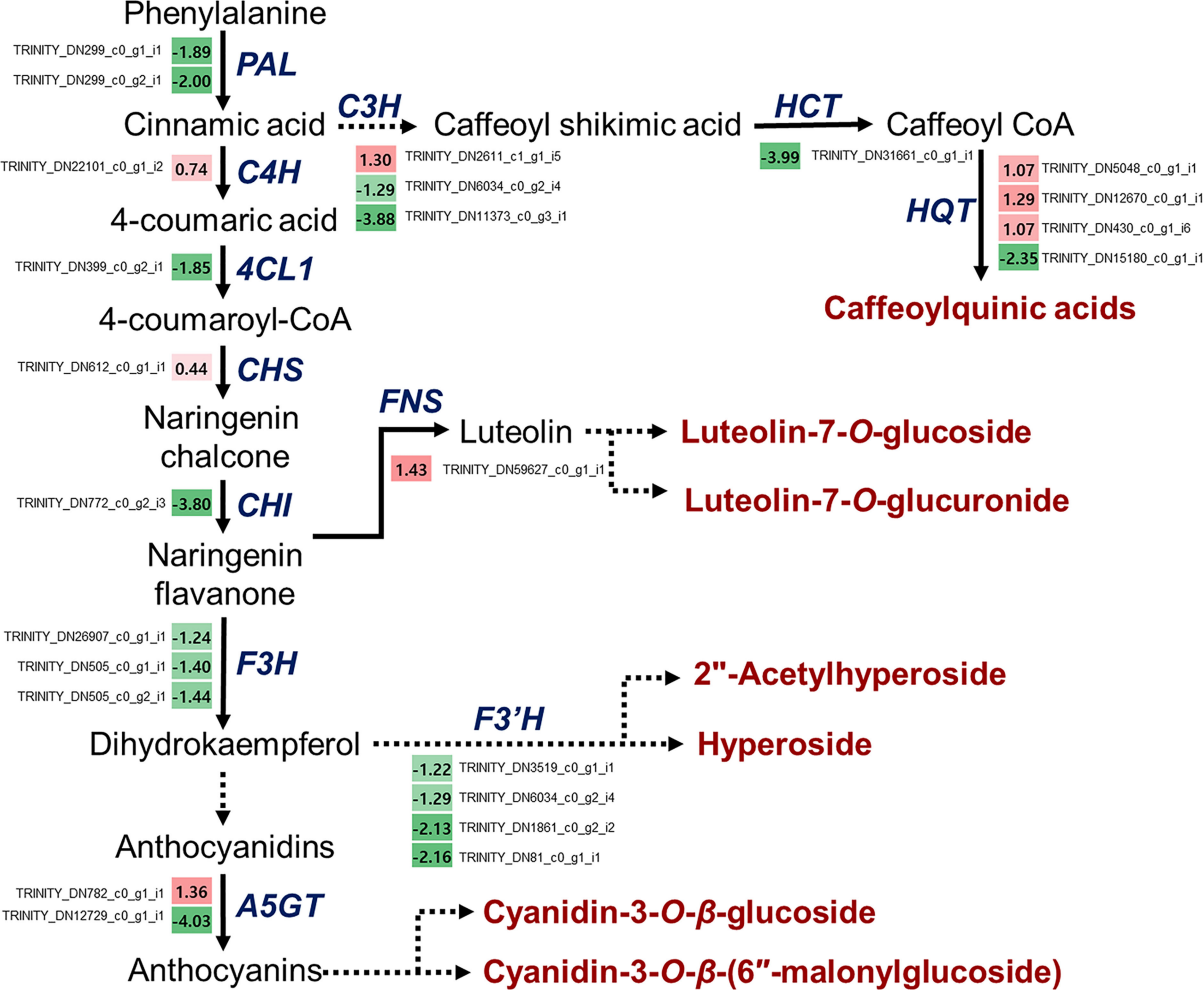
Drought-responsive genes in the phenylpropanoid biosynthesis from *L. fischeri*. A color gradient from low (green) to high (red) represents the relative levels of expression. The numbers in the squares indicate FC in gene expression in drought-induced *L. fischeri* plants, compared to control.

A schematic overview of the phenylpropanoid biosynthetic pathways and transcript levels of the genes involved in *L. fischeri* is shown in **Figure 4**. Also, fragments per kilobase of transcript per million mapped reads (FPKM) values obtained by RNA-Seq analysis were shown in **Supplementary Figure 2**. Among the 24 DEGs, 8 DEGs were upregulated, while 16 were downregulated under drought stress. In the initial step of phenylpropanoid biosynthesis, two DEGs of *LfPAL* (TRINITY_DN299_c0_g1_i1; log_2_FC=-0.89 and TRINITY_DN299_c0_g2_i1; log_2_FC=-2.00) were downregulated in *L. fischeri*. Moreover, one gene encoding *LfC4H* (TRINITY_DN22101_c0_g1_i2, log_2_FC=0.74) was specifically upregulated, whereas one gene encoding *Lf4CL* (TRINITY_DN399_c0_g2_i1, log_2_FC=-1.85) was downregulated under drought stress. Subsequently, we found that DEGs encoding *LfCHS* (TRINITY_DN612_c0_g1_i1, log_2_FC=0.44) and *LfCHI* (TRINITY_DN772_c0_g2_i3, log_2_FC=-3.80) were upregulated and downregulated in *L. fischeri* under drought stress, respectively. We isolated one gene encoding *LfFNS* (TRINITY_DN59627_c0_g1_i1, log_2_FC=1.43) that was significantly upregulated. However, three *LfF3H* (TRINITY_DN26907_c0_g1_i1; log_2_FC=-1.24, TRNITY_DN505_c0_g1_i1, log_2_FC=-1.40, and TRINITY_DN505_c0_g2_i1; log_2_FC=-1.44) and four *LfF3’H* (TRINITY_DN3519_c0_g1_i1; log_2_FC=-1.22, TRINITY_DN6034_c0_g2_i4; log_2_FC=-1.29, TRINITY_DN1861_c0_g2_i2; log_2_FC=-2.13, and TRINITY_DN81_c0_g1_i1; log_2_FC=-2.16) genes were downregulated under drought stress. Interestingly, two DEGs encoding *LfA5GT* were found to be upregulated (TRINITY_DN782_c0_g1_i1, log_2_FC=1.36) and downregulated (TRINITY_DN12729_c0_g1_i1, log_2_FC=-4.03) in *L fischeri*. Among the mapped DEGs related to hydroxycinamic acid biosynthesis, we obtained three genes encoding *LfC3H* (TRINITY_DN2611_c1_g1_i5; log_2_FC=1.30, TRINITY_DN6034_c0_g2_i4; log_2_FC=-1.29, and TRINITY_DN11373_c0_g3_i1; log_2_FC=-3.88), one gene encoding *LfHCT* (TRINITY_DN31661_c0_g1_i1, log_2_FC=-3.99), and four genes encoding *LfHQT* (TRINITY_DN5048_c0_g1_i1; log_2_FC=1.07, TRINITY_DN12670_c0_g1_i1, log_2_FC=1.29, TRINITY_DN430_c0_g1_i6; log_2_FC=1.07, and TRINITY_DN15180_c0_g1_i1; log_2_FC=-2.35) in *L. fischeri*.

### 3.5 Taxonomic analysis of *L. fischeri* transcriptome

**Figure 5:**
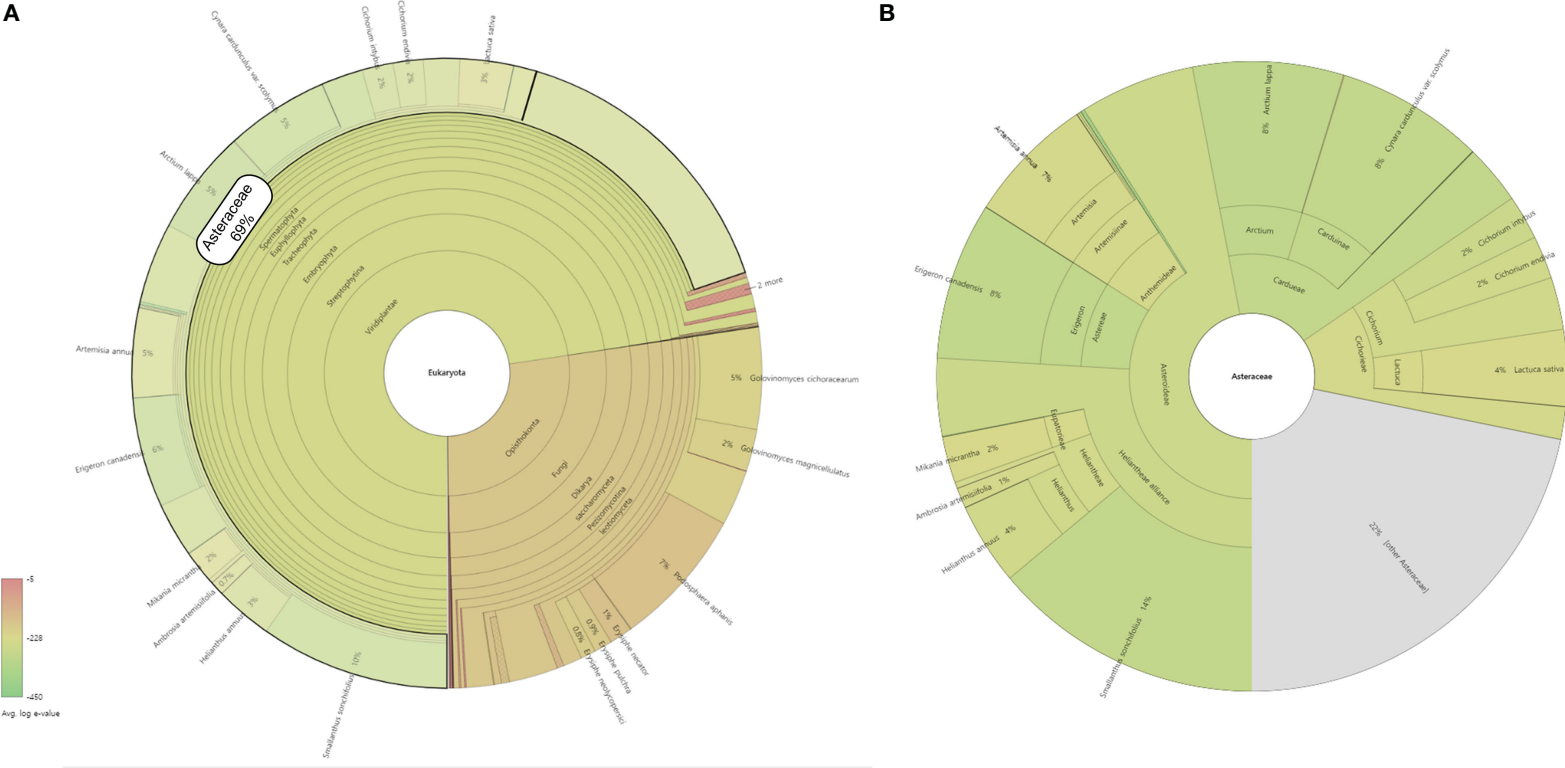
A taxonomic profile of BLASTP results against the NCBI NR database with the highest taxon as **(A)** Eukaryota and **(B)** Asteracea. The circles from inside to outside represent different taxonomic rankings, while the area of the sector indicates the fraction of various OUT annotation findings.

Translated sequences of 35,084 representative transcripts of *L. fischeri* were aligned against the NCBI NR protein database (released on Oct. 18, 2022) using default parameters. The taxonomic profile of the BLASTP results was visualized using the Krona Tools (v2.8.1) (Ondov et al., 2011). The majority of the hits (72%) were found in plant species, especially the ones belonging to the family Asteraceae (69%) (**Figure 5A**). In addition, various Asteraceae plants, such as *Smallanthus sonchifolius* (14%), *Erigeron canadensis* (8%), *Arctium lappa* (8%), *Cynara cardunculus* var. *scolymus* (8%) and *Artemisia annua* (7%) showed the highest taxonomic levels based on the BLASTP results against the NCBI NR database (**Figure 5B**). Of these, six species, *A. annua*, *C. cardunculus*, *E. canadensis*, *Helianthus annuus*, *Lactuca sativa*, and *Mikania micrantha*, which are frequently found in the BLASTP results, were selected to further investigate the distribution of key transcripts across the family Asteraceae (**Figure 4** and **Supplementary Table S5**) (Scaglione et al., 2016; Badouin et al., 2017; Reyes-Chin-Wo et al., 2017; Shen et al., 2018; Laforest et al., 2020; Liu et al., 2020).

The key transcripts shown in **Figure 4** were translated and searched against the proteome sequences of the six selected species belonging to the family Asteraceae using BLASTP. The same E-value criterion (< 1e-30) was used to filter out spurious hits (**Table 5** and **Supplementary Tables S5**). Interestingly, two transcripts were found to be *L. fischeri*-specific at the amino acid sequence level. For the transcript TRINITY_DN22101_c0_g1_i2 encoding *C4H*, there was only one hit from BLASTN searches, showing an alignment length of 297 bp found in *C. cardunculus* genome (E-value = 4.10e-74). For the transcript TRINITY_DN26907_c0_g1_i1 encoding *F3H*, BLASTN hits with short alignment lengths ranging from 113 bp to 126 bp were found in the genome sequences of *A. annua*, *E. canadensis*, *H. annuus*, and *M. micrantha*. This suggests that genes corresponding to the two transcripts are drought (or stress)-responsive and regulate the phenylpropanoid biosynthetic pathway. Notably, only one or two BLASTP hits were found in the proteomes of the six species for the translated transcript TRINITY_DN31661_c0_g1_i1. Thus, HCT plays a pivotal role in regulating the production of CQA and its derivatives in Asteraceae species.

**Table 5:** The number of BLASTP hits (E-value < 1e-30) of the translated transcripts shown in **Figure 4**.

Transcript	Symbol	Aa^*^	Cc^*^	Ec^*^	Ha^*^	Ls^*^	Mm^*^	Sum
TRINITY_DN299_c0_g1_i1	PAL	6	6	6	9	5	11	43
TRINITY_DN299_c0_g2_i1	PAL	6	7	6	9	5	12	45
TRINITY_DN22101_c0_g1_i2	C4H	0	0	0	0	0	0	0
TRINITY_DN399_c0_g2_i1	4CL	54	28	34	49	33	30	228
TRINITY_DN612_c0_g1_i1	CHS	15	10	11	23	12	13	84
TRINITY_DN772_c0_g2_i3	CHI	7	2	1	3	2	6	21
TRINITY_DN26907_c0_g1_i1	F3H	0	0	0	0	0	0	0
TRINITY_DN505_c0_g1_i1	F3H	124	97	101	143	113	76	654
TRINITY_DN505_c0_g2_i1	F3H	116	94	98	136	110	71	625
TRINITY_DN782_c0_g1_i1	A5GT	182	102	129	234	154	123	924
TRINITY_DN12729_c0_g1_i1	A5GT	213	124	155	293	174	164	1123
TRINITY_DN2611_c1_g1_i5	C3H	63	65	52	99	73	67	419
TRINITY_DN6034_c0_g2_i4	C3H	190	166	204	280	224	198	1,262
TRINITY_DN11373_c0_g3_i1	C3H	183	176	211	303	243	216	1,332
TRINITY_DN59627_c0_g1_i1	FNS	167	164	200	272	209	177	1,189
TRINITY_DN3519_c0_g1_i1	F3’H	321	189	229	341	265	254	1,599
TRINITY_DN6034_c0_g2_i4	F3’H	190	166	204	280	224	198	1,262
TRINITY_DN1861_c0_g2_i2	F3’H	199	176	221	317	249	216	1,378
TRINITY_DN81_c0_g1_i1	F3’H	312	186	228	340	264	252	1,582
TRINITY_DN31661_c0_g1_i1	HCT	1	1	1	1	1	2	7
TRINITY_DN5048_c0_g1_i1	HQT	42	15	32	55	13	29	186
TRINITY_DN12670_c0_g1_i1	HQT	22	10	19	36	7	15	109
TRINITY_DN430_c0_g1_i6	HQT	55	22	42	73	26	38	256
TRINITY_DN15180_c0_g1_i1	HQT	27	10	19	38	8	17	119

Aa (*Artemisia annua*), Cc (*Cynara cardunculus*), Ec (*Erigeron canadensis*), Ha (*Helianthus annuus*), Ls (*Lactuca sativa*), and Mm (*Mikania micrantha*). See **Table S1** for more information on the genomes used in this analysis.

## 4 Discussion

### 4.1 Metabolic response to drought stress in *L. fischeri*

In the present study, newly isolated flavones such as luteolin-7-*O*-*β*-glucoside (peak 3) and luteolin-7-*O*-*β*-glucuronide (peak 4), and anthocyanins, including cyanidin-3-*O*-*β*-glucoside (peak 9) and cyanidin-3-*O-β*-(6″-malonylglucoside) (peak 10), were considerably increased under drought stress in *L. fischeri*, while the most common phenolic compounds, called CQAs and flavonol content, were decreased. Compared to earlier findings, different metabolic responses have been revealed depending on plant, treatment. For instance, *Agave salmiana* under *in vitro* drought stress had the lowest levels of flavonols such as kaempferol glycosides and quercetin glycosides (Puente-Garza et al., 2017). Most of the polyphenols including luteolin-7-O-glycoside, 1,3-dicaffeoylquinic acid were upregulated with increasing drought stress duration in *Achillea pachycephala* Rech. f (Gharibi et al., 2019). These were similar to our finding. In contrast, the higher levels of flavonols were indicated under extreme drought stress in Arabidopsis. It has been investigated that flavonoids response to drought stress are variable, and the severity and length of the drought stress may have a significant impact on the types, quantities and localization of flavonoids in response to various levels of water shortage (Shojaie et al., 2016). The accumulation of phenolic acids and flavonoids is crucial for mitigating the deleterious effects of drought stress in plants. Enhanced levels of these compounds act as antioxidants and protect plants from detrimental effects of water scarcity. For instance, flavonoids, including kaempferol and quercetin, increased in tomato plants, similar to drought resistance. Flavonoid production in the cytoplasm may detoxify the detrimental H_2_O_2_ molecules caused by drought stress (Sharma et al., 2019). Additionally, flavonoid levels increased and showed that a water deficit had an impact on flavonoid accumulation, possibly by controlling hormone metabolism (Yuan et al., 2012). In our previous study, we observed that sunlight increased the total phenolic content in four weeks, whereas the total flavonoid content showed no significant change (Kim et al., 2012). Here, *L. fischeri* plants were grown under identical sunlight conditions and the water supply was removed. Compared to our present findings, the biosynthesis of hydroxycinnamic acids, such as CQA and its derivatives, may be positively affected by sunlight but negatively by drought stress.

Plant cultivars with high anthocyanin levels tend to be more tolerant to drought conditions (Cirillo et al., 2021). Drought reduces grain weight and substantially decreases anthocyanin, protein, and carbohydrate content in *Triticum aestivum* L. cv. Guizi 1 is a drought-tolerant purple wheat cultivar (Li et al., 2020). Plant leaves synthesize anthocyanins for adaptive purposes, such as photoprotection, cold hardiness, and antioxidative capabilities (Archana et al., 2020). Drought tolerance is an adaptive response (Egilla et al., 2001). It has been proposed that anthocyanins operate as osmoregulators by controlling water homeostasis in stressed plants. These compounds build up in tissues under water shortage, and tissues with higher anthocyanin levels are more drought-resistant. It is well known that anthocyanins can remove reactive oxygen species (ROS) and transport them to vacuoles (Zahedi et al., 2021). Similar to other Asteraceae species, safflower (*Carthamus tinctorius*) responded to drought stress by producing significantly greater levels of total flavonoid, anthocyanins (Toupchi Khosrowshahi et al., 2019). Also, water stress reduced chlorophyll content while increasing flavonoids, anthocyanin, phenolic compounds, and soluble sugars in *Helianthus annuus* and *Silybrum marianum* (Ebrahimian and Bybordi, 2012). Therefore, the pronounced induction of anthocyanins in *L. fischeri* leaves could be viewed as a physiological response to water deficits.

### 4.2 Drought-induced transcriptome responses classified by functional categories

Transcriptome sequencing is an effective approach to identify the molecular mechanisms underlying plant responses to several abiotic stressors. In a variety of crops, genome-scale transcriptome studies based on Illumina RNA Sequencing have been used to assess gene expression under cold, drought, and heat stresses (Qiu et al., 2017). In addition, identifying DEGs across treatments/conditions is an important phase, and frequently the primary objective, in statistical RNA-Seq data analysis. The discovery of DEGs contributes in the understanding of gene function when cells react to various chemo. Furthermore, finding DEGs can be used as a preliminary to grouping gene expression profiles or assessing gene set enrichments (Kvam et al., 2012). Several studies have been conducted on drought stress in various crops, such as wheat, sweet potato, maize, and rice, using transcriptome analysis (Zhao et al., 2021). To the best of our knowledge, this is the first RNA-Seq study of *L. fischeri* in drought response analysis. Therefore, this study provides important information on drought-responsive processes at the transcriptional level.

Various drought-responsive genes have been identified using RNA-Seq analysis in *L. fischeri*. Most of these genes were found to be involved in phytohormone signaling, MAPK and calcium signaling pathways, transcription factors regulating gene expression, and other drought-inducible regulatory processes. Phytohormones, including ABA, auxin, cytokinin, ethylene, gibberellic acid, and jasmonic acid mediate a variety of activities and enable plants to withstand drought (Wilkinson et al., 2012). ABA is an essential player that controls physiological and molecular reactions to water deficit, including gene expression, osmoprotectants, stomatal closure, and stress protein production (Takahashi et al., 2018). According to our results, eight transcripts (TRINITY_DN22327_c0_g1_i1, TRINITY_DN1659_c0_g1_i3, TRINITY_DN4970_c1_g1_i2, TRINITY_DN14995_c0_g1_i1, TRINITY_DN1678_c0_g1_i2, TRINITY_DN17252_c0_g1_i1, TRINITY_DN73142_c0_g1_i1, and TRINITY_DN15780_c0_g3_i1) matched with 13 query proteins that showed the highest upregulation rates under drought stress in *L. fischeri* (**Supplementary Table 6**). Interestingly, all of these transcripts were associated with ABA signaling. In addition, many DEGs were annotated and displayed various distributions in the MAPK signaling pathway and transcription factors under drought stress. Upon drought stimulation, phytohormone levels typically increase, activating morphophysiological and other biochemical pathways. These pathways may consist of the calcium signaling pathway, MAPK signaling pathway, regulation of transcription factors, and higher levels of antioxidant enzymes (Ilyas et al., 2021).

### 4.3 Phenylpropanoid biosynthesis under drought stress

Plants have developed complex molecular, physiological, and biochemical processes to deal with the consequences of drought (Zhao et al., 2021). Physiological reactions include stomatal closure, decreases in photosynthesis rate, effects on photosynthetic proton and electron transport, and modifications to photosynthetic carbon reduction and carbon oxidation cycles when there is a shortage of water in the soil (Cheng et al., 2018). Additionally, drought stress can affect secondary metabolic systems, and various findings on secondary metabolite formation in medicinal plants under drought stress have been investigated (Yadav et al., 2014; Zahir et al., 2014a; Jia et al., 2015).

Phenolic compounds are the most abundant secondary metabolites in plants, with simple to complex aromatic rings. They are classified into various classes including phenolic acids, flavonoids, stilbenes, and lignans, each of which has unique qualities. Because these compounds are derived from phenylalanine, they are also referred to as phenylpropanoids (Sharma et al., 2019). In the initial step of the phenylpropanoid biosynthetic pathway, *PAL* exists at the interface between the primary and secondary metabolism to convert L-phenylalanine to cinnamic acid (Pappi et al., 2021). The subsequent stages of the pathway, catalyzed by *C4H* and *4CL*, are required and serve as the basis for all further branches and resulting metabolites (Deng & Lu, 2017). In this study, we isolated one gene encoding *LfFNS* which was significantly upregulated under drought stress. This result likely explains the increased flavone content, including luteolin-7-*O*-glucoside and luteolin-7-*O*-glucuronide, in *L. fischeri* under drought stress. In contrast, three *LfF3H* and four *LfF3’H* genes were considerably downregulated under drought stress. This may decrease the content of flavonols, including hyperoside and 2″-acetylhyperoside, in *L. fischeri*. These findings are similar to those of previous studies. For instance, most of the flavonoid concentrations, including apigenin, caffeic acid, chlorogenic acid, luteolin-7-*O*-glycoside, luteolin, kaempferol, 1,3-dicaffeoylquinic acid, and rutin, and the expression of the relevant genes, such as *PAL*, *CHS*, *CHI*, *F3H*, *F3’H*, *F3’5’H*, and *FLS*, also increased after 21 days of exposure to drought in *Achillea pachycephala* Rech.f (Gharibi et al., 2019). Previously, it was also discovered that genes involved in flavonoid production were increased in buckwheat during drought stress (Hou et al., 2019). Anthocyanins are glycosides of anthocyanidins, and their glycosylation is carried out by a variety of enzymes, including UDP-glucose and anthocyanidin 3-*O*-glucosyltransferase (UF3GT), which is the most well-studied flavonoid glycosyltransferase (Deng and Lu, 2017). Based on our results, *LfA5GT* was upregulated under drought stress, which might explain the newly identified and enhanced anthocyanin content in *L. fischeri*. This is also in accordance with previous findings that an increase in the overall anthocyanin, flavonoid, and phenolic content may be closely related to the enhanced levels of flavonoid biosynthetic genes, including *CHS*, *CHI*, *F3H*, *FNS*, *FLS*, *DFR*, and *ANS*, in wheat leaves under drought stress (Ma et al., 2014).

The presence of a benzene ring bonded to one or more hydroxyl or methoxy groups distinguishes phenolic acids from aromatic acids. These compounds can be classified into two classes based on their constitutive carbon skeletons: hydroxycinnamic acid and hydroxybenzoic acid. The major compounds in *L. fischeri* and the CQAs belong to the hydrocinnamic acid group. Although phenolic acids are abundantly dispersed in plants, it has been established that they are produced *via* the shikimate pathway; however, they are still unknown and have been revised (Deng and Lu, 2017). Three biosynthetic steps have been suggested for CQA formation in plants. The first involves caffeic acid coenzyme A and quinic acid catalysis by *HQT*. The second involves the production of CQAs by employing caffeoyl glucoside as an active intermediate, which is catalyzed by hydroxyl cinnamoyl D-glucose:quinate hydroxycinnamoyl transferase (*HCGQT*). Finally, CQAs were synthesized from *p*-coumaroyl quinic acid *via* a catalytic reaction involving *HCT* and *C3H*. Notably, the *HQT*-mediated pathway is the most important for CQA biosynthesis among the three pathways (Xu et al., 2022). The transcript levels of two *LfC3H* genes, *LfHCT* and *LfHQT* were downregulated only under drought stress in *L. fischeri*. These findings were consistent with the decreased levels of CQA and its derivatives.

Drought stress has a negative impact on both the quantitative and qualitative aspects of growth, production, and agricultural output. It interferes with the crop’s normal physiological functioning by reducing nutrient delivery and causing cellular toxicity. It aslo leads to membrane destabilization, harm to the photosynthetic apparatus, and oxidative stress (Yadav et al., 2021). Plants undergo considerable molecular and physiological changes as a result of drought stress, and global transcriptional regulation is regarded as the most basic molecular response of plants to adapt to and deal with drought stress (Qiu et al., 2017). These changes include the transcriptomic, proteomic and metabolomics modifications of plants, which leads to cellular biosynthesis and breakdown activities. To maintain growth and yield during water shortage, thus, plants activate various strategies such as increased synthesis of secondary metabolites, phytohormones, ROS signaling, plant hydraulic status, and osmotic adjustment (Jogawat et al., 2021). Of these, the biosynthesis of secondary metabolites induced by drought stress has been investigated. Flavonoids and polyphenols are well known as adaptive natural substances that enable to plants to scavenge ROS under drought stress (Treml and Šmejkal, 2016). As a consequence, numerous crop plants were shown to have enhanced biosynthetic pathways associated with their accumulations. A variety of biotechnological and bioinformatics methods, including transcriptomics, metabolomics, proteomics, have also been demonstrated to significantly stimulate these metabolites between drought-toleranct and drought-sensitive varieties or cultivars (Yadav et al., 2021). In previous study, expression level of 4CL, a key gene in the catechin production, was decreased by drought treatment in *Camellia sinensis*. It suggested that increased polyphenol accumulation, including isoflavonoids and catechins, is related to resistance in this plant (Cheruiyot et al., 2008). In potato, expression levels of genes including flavonone-3-hydroxylase, flavonol synthase, and *β*-carotene synthase, which are crucial for the flavonoids, carotenoids, and other phenolic compounds production, were enhanced during drought stress. The transcript levels of these genes impacts how well some potato cultivars tolerate drought (Min et al., 2008). Moreover, it was discovered that flavonoid biosynthetic genes, as well as genes linked to lignin, were induced in response to drought stress in cotton. This ultimately results in the polyphenols and xanthophylls accumulation (Ranjan et al., 2012). Above this, various plants have indicated varied increases in flavonoids and polyphenols or the gene expression involved in their biosynthesis under water deficit (Yadav et al., 2021). Based on our findings (**Table 2** and **Figure 4**), we suggest that the upregulated *LfFNS* (TRINITY_DN31661_c0_g1_i1) and *LfA5GT1* (TRINITY_DN782_c0_g1_i1), which are associated with the biosynthesis of flavones and anthocyanins, respectively, are candidate drought-responsive genes in *L. fischeri*. In addition, we observed two downregulated genes, *LfHCT* (TRINITY_DN31661_c0_g1_i1) and *LfHQT4* (TRINITY_DN15180_c0_g1_i1), which led to a decrease in CQAs and may play a crucial role in drought response in *L. fischeri*.

In summary, the drought response in *L. fischeri* is a complicated process that involves several morphological and molecular changes at all levels. Two flavones and two anthocyanins were newly isolated and were significantly elevated under drought stress. In contrast, the contents of CQAs and flavonols was decreased. These results indicate that the biosynthesis of CQAs and flavonols was downregulated, whereas flavones and anthocyanin biosynthesis were upregulated in response to drought stress. Furthermore, we used RNA-Seq analysis to assess transcriptional changes to obtain insights into the molecular response to drought stress in *L. fischeri*. A total of 2,105 hits for 516 different transcripts were found to be drought-responsive genes, and their expression levels were distributed in intricate patterns, spanning up- and downregulation under drought stress. Additionally, KEGG enrichment analysis showed that DEGs involved in phenylpropanoid biosynthesis were found in the highest number of both up-and downregulated DEGs. In particular, several DEGs were associated with flavonoid biosynthesis. Based on the regulation of phenylpropanoid biosynthetic genes, we propose that the upregulated *LfFNS* (TRINITY_DN31661_c0_g1_i1) and *LfA5GT1* (TRINITY_DN782_c0_g1_i1) genes are potential drought-responsive genes that correspond to the high concentrations of flavones and anthocyanins in *L. fischeri* under drought stress. Moreover, the downregulated genes, *LfHCT* (TRINITY_DN31661_c0_g1_i1) and *LfHQT4* (TRINITY_DN15180_c0_g1_i1), which resulted in a decrease in CQAs, may play a key role in the *L. fischeri* drought response. In particular, only one or two BLASTP hits for the translated transcript *LfHCT* (TRINITY_DN31661_c0_g1_i1) were obtained in the proteomes of the different Asteraceae species. This could imply that the HCT gene is important for the regulation of CQA and CQA derivatives in this species. In addition, it is interesting that two transcripts were identified as being *L. fischeri*-speicific at the amino acid sequence level, such as TRINITY_DN22101_c0_g1_i2 and TRINITY_DN26907_c0_g1_i1 encoding *LfC4H* and *LfF3H*, respectively. These findings contribute to a better understanding of drought-responsive changes in morphological, physiological, biochemical, and molecular processes, specifically the regulatory mechanisms of certain genes involved in phenylpropanoid biosynthesis in mitigating drought severity in *L. fischeri*.

## Data availability statement

The datasets presented in this study can be found in online repositories. The names of the repository/repositories and accession number(s) can be found below: https://www.ncbi.nlm.nih.gov/, SRR11585851; https://www.ncbi.nlm.nih.gov/, SRR11585852; https://www.ncbi.nlm.nih.gov/, SRR11585853; https://www.ncbi.nlm.nih.gov/, SRR11585854; https://www.ncbi.nlm.nih.gov/, SRR11585855; https://www.ncbi.nlm.nih.gov/, SRR11585856.

## Author contributions

SMK contributed to the conception and design of the study. YJP and DYK performed the experiments and analyzed the data. YJP and DYK drafted the manuscript. S-CH and JC performed the bioinformatics analysis. SYK, TQT, and JM critically reviewed the manuscript and helped with the data interpretation. SMK supervised the research project and the experiments. All authors contributed to the article and approved the submitted version.
