# Supplementary material for: Identification of drought-responsive phenolic compounds and their biosynthetic regulation under drought stress in Ligularia fischeri
Source: Front Plant Sci. 2023 Feb 13;14:1140509. doi: 10.3389/fpls.2023.1140509 (PMC9968736; doi:10.3389/fpls.2023.1140509)
Supplement: Supplementary file 1 [file DataSheet_1.docx]

Supplementary Material

Identification of drought-responsive phenolic compounds and their biosynthetic regulation under drought stress in *Ligularia fischeri*

Yun Ji Park, Do Yeon Kwon, Song Yi Koo, To Quyen Truong , Sung-Chul Hong, Jaeyoung Choi, Jinyoung Moon, Sang Min Kim^*^

*** Correspondence: Sang Min Kim:** [**kimsm@kist.re.kr**](mailto:kimsm@kist.re.kr)

## Supplementary Figures


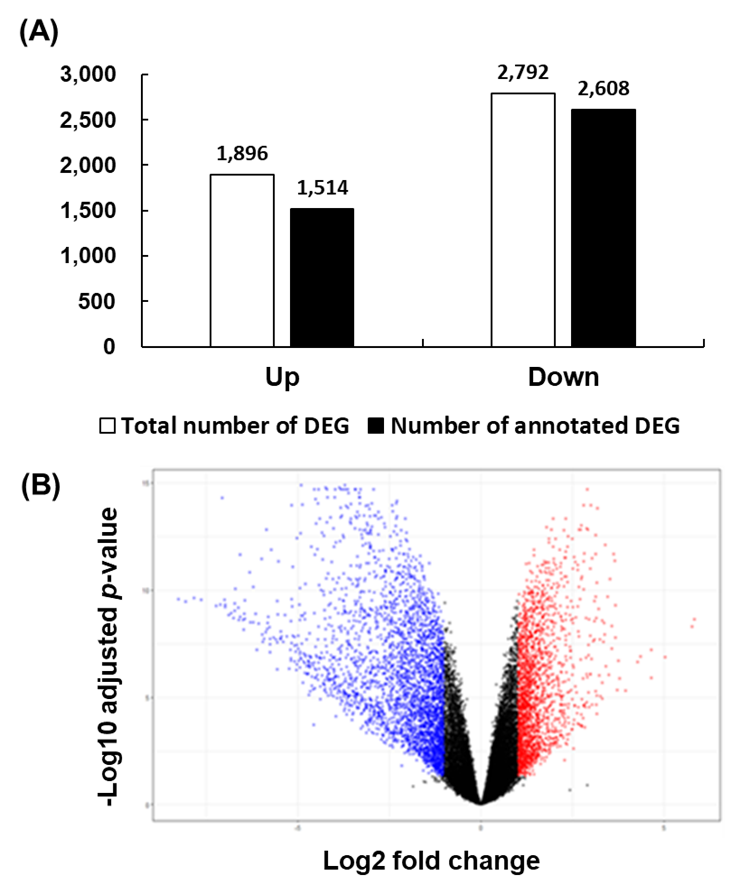


**Supplementary Figure 1.** DEGs in response to drought stress. (A) The number of up- and down-regulated DEGs during drought treatment. In the bar graph, white and black colors are shown as total number of DEGs and the number of annotated DEGs against NR database, respectively. (B) Volcano plot. The x-axis represents log of fold change and the y-axis indicates statistical significance. Blue dots show downregulated DEGs and red dots reveal upregulated DEGs. Black dots indicate not significant genes out of the filter criteria.


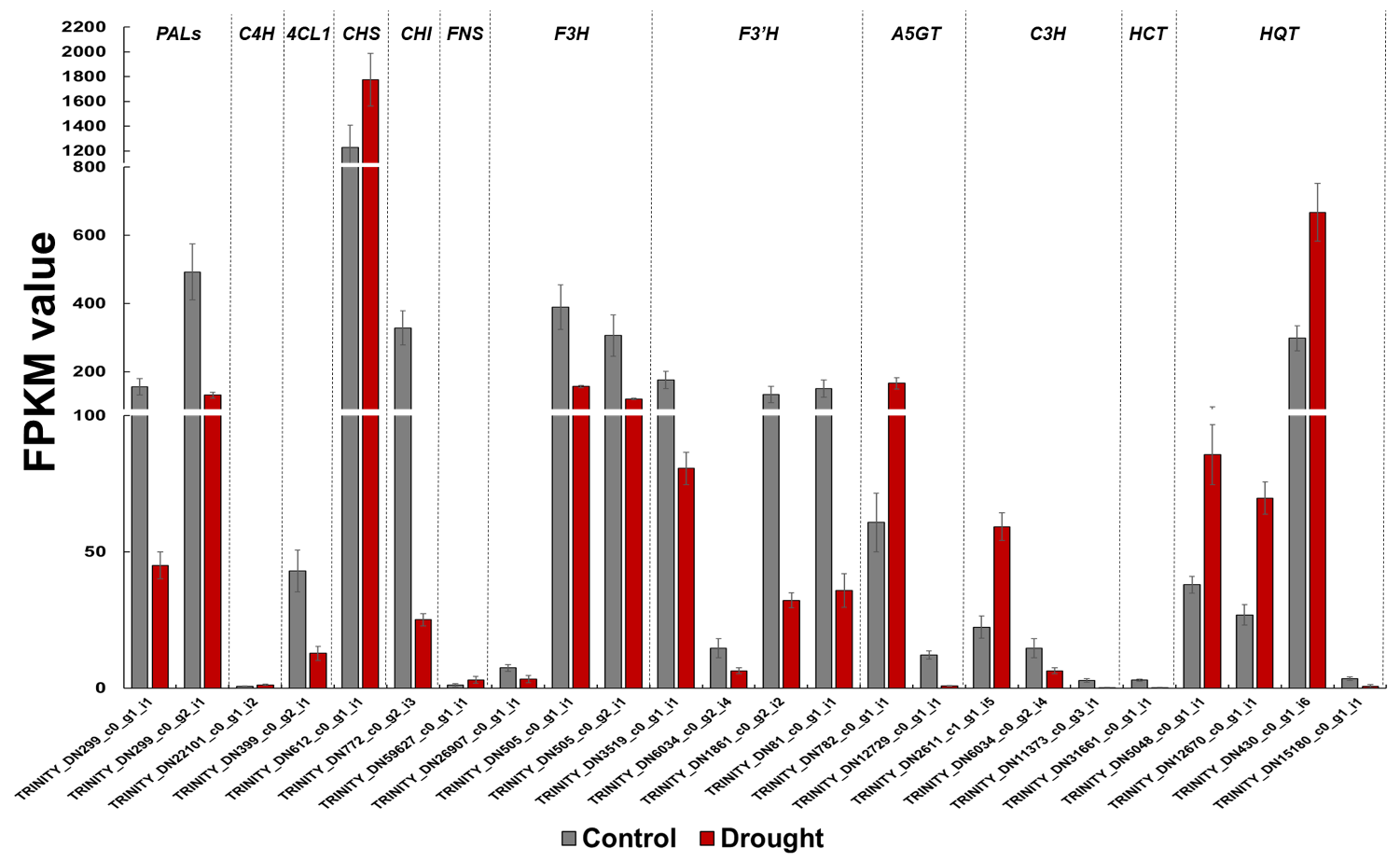


**Supplementary Figure 2.** FPKM values for 24 meaningful phenylpropanoid biosynthetic genes in *L. fischeri* under drought stress.

**Supplementary Table 1.** Summary of *L. fischeri* transcriptome.

| Sample | No. raw reads | No. clean reads | No. mapped reads | Mapping rate (%) |
| --- | --- | --- | --- | --- |
| Control | 18,173,786 | 17,333,040 | 16,331,427 | 94.22 |
|  | 16,812,210 | 16,015,930 | 14,821,407 | 92.54 |
|  | 14,168,312 | 13,556,017 | 12,631,512 | 93.18 |
| Drought | 15,081,088 | 14,459,649 | 13,757,094 | 93.88 |
|  | 14,390,377 | 13,677,518 | 12,889,623 | 94.24 |
|  | 15,746,649 | 14,998,725 | 13,938,083 | 92.93 |

**Supplementary Table 2.** Summary of assembled gene sets

| Data | No. transcripts | Sum.of bp | Length(bp) of transcripts | | | |
| --- | --- | --- | --- | --- | --- | --- |
|  |  |  | Min | Max | Aver. | N50* |
| Total transcripts | 74,873 | 99,080,462 | 500 | 14,394 | 1,323 | 1,583 |
| Representative transcripts | 35,084 | 42,015,840 | 500 | 14,394 | 1,197 | 1,424 |

* a weighted median statistic length such that 50% of the assembled transcripts are longer than the N50 length

**Supplementary Table 3.** Categorization of drought-responsive processes and relevant protein query information.

| Category | Relevant functional group | Accession No. of protein sequence |
| --- | --- | --- |
| **ABA signaling** | Pyrabactin resistance 1 (PYR)/PYR1-like (PYL)/regulatory component of ABA receptor (RCAR) | O49686.1 Q8VZS8.1 AEE27277.1 AED96316.1 |
|  | Protein phosphatase 2C | NP_001331640.1 NP_001119448.1 NP_200515.1 |
|  | Sucrose nonfermenting 1 (SNF1)-related protein kinase 2 | AED91326.1 AEE78672.1 AED98274.1 AEE33750.1 |
|  | ABRE-binding proteins (AREBs)/ABRE-binding factors (ABFs) | NP_564551.1 NP_001320130.1 NP_566629.1 |
|  | DRE-binding proteins (DREBs) | NP_196160.1 |
|  | AtABCG25 | NP_565030.1 |
|  | AtABCG40 | Q9M9E1.1 |
|  | AtABCG31 | Q7PC88.1 |
|  | AtABCG30 | Q8GZ52.2 |
|  | AIT1 (nitrate transporter 1:2) | NP_564978.1 |
|  | Nitrate transporter 1 (NRT1/PTR) | NP_563899.1 |
|  | Peptide transporter | AAN13027.1  BAB08249.1  BAA97215.1 |
|  | DTX50, ABA exporter | Q9FJ87.1 |
| **Phosphorylation** | ABA-insensitive 3 | BAB01214.1 |
|  | ABA-insensitive 5 | Q9SJN0.1 |
|  | Brassinosteroid (BR)-insensitive 2 | Q39011.2 |
| **Osmotic stress sensing** | Histidine kinase | AAA70047.1 OAP07328.1 NP_568532.1 NP_564276.1 Q9C5U0.1 Q9SXL4.2 |
|  | Reduced hyperosmolality-induced Ca2+ increase 1 | Q9XEA1.1 |
| **MAPK and Calcium signaling** | Mitogen-activated protein kinase | BAA09057.1 BAB02403.1 |
|  | MAPK kinase 3 | O80396.1 |
|  | MAPKKK17/18-MKK3-MPK1/2/7/14 | NP_180810.1 NP_172003.1 NP_001320530.1 NP_974049.1 NP_179409.1 NP_195363.1 |
|  | ABA-insensitive 1 | P49597.2 |
|  | Calcium-dependent protein kinase (CPK4, CPK11, CPK12, CPK32) | NP_192695.1 NP_174807.1 NP_197748.1 NP_191312.2 |
|  | bZIP-type transcription factors | NP_849777.1 |
| **Transcription factors regulating gene expression during drought stress** | APETALA2/Ethylene-responsive-binding factor (AP2/ERF) family | NP_196160.1 |
|  | WRKY transcription factor (WRKY18, WRKY40, WRKY60) | NP_567882.1 NP_178199.1 NP_180072.1 |
|  | ABA overly sensitive 3/WRKY63 | NP_001319203.1 |
|  | NAC (NAM-ATAF1,2-CUC2) transcription factor (ANAC096, ANAC055, ANAC019, ANAC072/RD26, ANAC002/ATAF1, ANAC081/ATAF2, ANAC102, ANAC032) | NP_199471.1 NP_188169.1 NP_175697.1 Q93VY3.1 OAO97067.1 Q39013.2 OAP14514.1 Q9C598.1 NP_001330407.1 NP_177869.1 |
|  | ANAC016 | NP_001077648.1 |
|  | MYB96 | NP_201053.2 |
|  | MYC2 | NP_174541.1 |
|  | Arabidopsis Homologue of Trithorax 1 | NP_850170.1 |
| **Epigenetic regulation  (DNA methylation, histone modification)** | Histone deacetylase 9 | NP_190054.2 |
| **Drought stress-inducible genes  encoding functional proteins** | Superoxide, ascorbate peroxidase, Arabidopsis glutathione peroxidase | CAA66640.1 CAA61965.1 AAC09173.1 CAA04112.1 |
|  | Sugar- or Proline-biosynthetic enzymes, galactinol synthase 2 | NP_176053.1 |

**Supplementary Table 4.** Gene list of DEGs categorized as “response to water deprivation” and “flavonoid metabolic process” based on GO analysis of *L. fischeri* transcriptome data under drought stress.

| GO Name | Response to water deprivation | | Flavonoid metabolic process | |
| --- | --- | --- | --- | --- |
| GO ID | GO:0009414 | | GO:0009812 | |
| No. | Up | Down | Up | Down |
| 1 | TRINITY_DN2417_c0_g1_i2 | TRINITY_DN225_c0_g1_i2 | TRINITY_DN18561_c0_g1_i2 | TRINITY_DN12729_c0_g1_i1 |
| 2 | TRINITY_DN3812_c0_g1_i1 | TRINITY_DN5_c0_g2_i1 | TRINITY_DN12670_c0_g1_i1 | TRINITY_DN5173_c0_g1_i2 |
| 3 | TRINITY_DN153_c0_g1_i2 | TRINITY_DN1991_c0_g1_i2 | TRINITY_DN780_c0_g2_i1 | TRINITY_DN3063_c0_g1_i1 |
| 4 | TRINITY_DN1255_c0_g4_i1 | TRINITY_DN51239_c0_g1_i1 | TRINITY_DN2417_c0_g1_i2 | TRINITY_DN6433_c0_g2_i4 |
| 5 | TRINITY_DN3919_c0_g1_i1 | TRINITY_DN6011_c0_g1_i16 | TRINITY_DN14679_c0_g1_i2 | TRINITY_DN772_c0_g2_i3 |
| 6 | TRINITY_DN13686_c0_g1_i1 | TRINITY_DN10726_c0_g1_i1 | TRINITY_DN868_c0_g1_i1 | TRINITY_DN13113_c0_g1_i1 |
| 7 | TRINITY_DN14714_c0_g1_i2 | TRINITY_DN9645_c0_g1_i4 | TRINITY_DN32961_c0_g1_i1 | TRINITY_DN6120_c0_g2_i1 |
| 8 | TRINITY_DN8948_c0_g1_i1 | TRINITY_DN56980_c0_g1_i1 | TRINITY_DN29911_c0_g4_i3 | TRINITY_DN15112_c0_g2_i1 |
| 9 | TRINITY_DN17661_c0_g1_i2 | TRINITY_DN1191_c0_g2_i1 | TRINITY_DN183_c0_g1_i5 | TRINITY_DN12741_c0_g1_i1 |
| 10 | TRINITY_DN1068_c0_g1_i3 | TRINITY_DN4685_c0_g1_i2 | TRINITY_DN15081_c0_g1_i1 | TRINITY_DN11768_c0_g2_i1 |
| 11 | TRINITY_DN4061_c0_g1_i2 | TRINITY_DN24626_c0_g1_i1 | TRINITY_DN8522_c0_g3_i1 | TRINITY_DN26247_c0_g1_i1 |
| 12 | TRINITY_DN7191_c0_g1_i1 | TRINITY_DN1771_c0_g1_i2 | TRINITY_DN5048_c0_g1_i1 | TRINITY_DN16030_c0_g1_i1 |
| 13 | TRINITY_DN186_c0_g1_i5 | TRINITY_DN5621_c0_g1_i1 | TRINITY_DN17211_c0_g1_i2 | TRINITY_DN299_c0_g1_i1 |
| 14 | TRINITY_DN13401_c0_g1_i1 | TRINITY_DN4121_c0_g1_i1 | TRINITY_DN5048_c0_g2_i1 | TRINITY_DN3519_c0_g1_i1 |
| 15 | TRINITY_DN3644_c0_g1_i1 | TRINITY_DN3112_c0_g1_i6 | TRINITY_DN1039_c1_g1_i7 | TRINITY_DN14514_c0_g1_i2 |
| 16 | TRINITY_DN322_c1_g1_i1 | TRINITY_DN5571_c0_g1_i2 | TRINITY_DN15322_c0_g1_i2 | TRINITY_DN2677_c0_g2_i2 |
| 17 | TRINITY_DN3310_c0_g1_i1 | TRINITY_DN5208_c0_g1_i18 | TRINITY_DN430_c0_g1_i6 | TRINITY_DN18478_c0_g1_i1 |
| 18 | TRINITY_DN13628_c0_g1_i1 | TRINITY_DN2786_c0_g1_i1 | TRINITY_DN886_c0_g1_i1 | TRINITY_DN20146_c0_g1_i2 |
| 19 | TRINITY_DN4887_c0_g1_i1 | TRINITY_DN948_c0_g1_i4 | TRINITY_DN6816_c0_g1_i1 | TRINITY_DN16863_c0_g2_i1 |
| 20 | TRINITY_DN15427_c0_g1_i4 | TRINITY_DN13284_c0_g1_i2 | TRINITY_DN1759_c0_g1_i3 | TRINITY_DN13662_c0_g1_i3 |
| 21 | TRINITY_DN14335_c0_g1_i1 | TRINITY_DN4599_c0_g1_i1 | TRINITY_DN6283_c0_g1_i3 | TRINITY_DN399_c0_g2_i1 |
| 22 | TRINITY_DN14195_c0_g1_i1 | TRINITY_DN16092_c0_g1_i1 | TRINITY_DN395_c0_g2_i1 | TRINITY_DN1352_c0_g1_i2 |
| 23 | TRINITY_DN1825_c0_g1_i2 | TRINITY_DN3312_c0_g1_i1 | TRINITY_DN395_c0_g1_i1 | TRINITY_DN17882_c0_g1_i2 |
| 24 | TRINITY_DN1547_c0_g2_i2 | TRINITY_DN31828_c0_g1_i5 |  | TRINITY_DN24248_c0_g1_i1 |
| 25 | TRINITY_DN4396_c0_g1_i1 | TRINITY_DN1698_c2_g2_i1 |  | TRINITY_DN63870_c0_g1_i1 |
| 26 | TRINITY_DN4816_c0_g1_i1 | TRINITY_DN26276_c0_g1_i2 |  | TRINITY_DN6968_c0_g1_i2 |
| 27 | TRINITY_DN2442_c0_g1_i2 | TRINITY_DN299_c0_g1_i1 |  | TRINITY_DN2239_c0_g1_i1 |
| 28 | TRINITY_DN1001_c0_g1_i2 | TRINITY_DN19734_c0_g1_i1 |  | TRINITY_DN4708_c0_g1_i1 |
| 29 | TRINITY_DN8033_c0_g1_i5 | TRINITY_DN835_c0_g1_i2 |  | TRINITY_DN13743_c0_g1_i3 |
| 30 | TRINITY_DN15196_c0_g1_i1 | TRINITY_DN1305_c0_g1_i4 |  | TRINITY_DN10372_c0_g1_i1 |
| 31 | TRINITY_DN724_c0_g2_i3 | TRINITY_DN5571_c0_g2_i1 |  | TRINITY_DN299_c0_g2_i1 |
| 32 | TRINITY_DN23668_c0_g1_i1 | TRINITY_DN15580_c0_g1_i3 |  | TRINITY_DN689_c0_g1_i3 |
| 33 | TRINITY_DN1090_c0_g2_i1 | TRINITY_DN5111_c1_g2_i2 |  | TRINITY_DN52191_c0_g1_i1 |
| 34 | TRINITY_DN11528_c0_g1_i1 | TRINITY_DN17882_c0_g1_i2 |  | TRINITY_DN21450_c0_g1_i1 |
| 35 | TRINITY_DN4887_c0_g2_i2 | TRINITY_DN9596_c0_g1_i1 |  | TRINITY_DN20842_c0_g2_i1 |
| 36 | TRINITY_DN18311_c0_g3_i1 | TRINITY_DN5111_c0_g1_i9 |  | TRINITY_DN26907_c0_g1_i1 |
| 37 | TRINITY_DN22327_c0_g1_i1 | TRINITY_DN3806_c0_g3_i1 |  | TRINITY_DN31291_c0_g2_i2 |
| 38 | TRINITY_DN13469_c0_g1_i3 | TRINITY_DN916_c0_g2_i1 |  | TRINITY_DN5496_c0_g1_i2 |
| 39 |  | TRINITY_DN2512_c0_g1_i10 |  | TRINITY_DN19359_c0_g1_i1 |
| 40 |  | TRINITY_DN3499_c0_g1_i1 |  | TRINITY_DN11109_c0_g5_i1 |
| 41 |  | TRINITY_DN6710_c0_g2_i2 |  | TRINITY_DN1154_c0_g1_i1 |
| 42 |  | TRINITY_DN1503_c0_g1_i3 |  | TRINITY_DN76_c0_g1_i1 |
| 43 |  | TRINITY_DN299_c0_g2_i1 |  | TRINITY_DN11534_c0_g1_i1 |
| 44 |  | TRINITY_DN3956_c0_g1_i1 |  | TRINITY_DN19590_c0_g1_i1 |
| 45 |  | TRINITY_DN3450_c0_g2_i6 |  | TRINITY_DN505_c0_g2_i1 |
| 46 |  | TRINITY_DN17173_c0_g1_i1 |  | TRINITY_DN17944_c0_g1_i4 |
| 47 |  | TRINITY_DN5536_c0_g1_i6 |  | TRINITY_DN4701_c4_g1_i1 |
| 48 |  | TRINITY_DN163_c1_g2_i1 |  | TRINITY_DN505_c0_g1_i1 |
| 49 |  | TRINITY_DN8995_c0_g1_i2 |  | TRINITY_DN6260_c0_g2_i3 |
| 50 |  | TRINITY_DN5571_c0_g3_i1 |  | TRINITY_DN4035_c0_g1_i2 |
| 51 |  | TRINITY_DN10902_c0_g2_i1 |  | TRINITY_DN11279_c0_g1_i1 |
| 52 |  | TRINITY_DN14791_c0_g1_i1 |  | TRINITY_DN24221_c0_g1_i1 |
| 53 |  | TRINITY_DN44796_c0_g1_i1 |  | TRINITY_DN6120_c0_g1_i1 |
| 54 |  | TRINITY_DN916_c0_g1_i5 |  | TRINITY_DN11339_c0_g1_i3 |
| 55 |  | TRINITY_DN14246_c0_g1_i2 |  | TRINITY_DN6587_c0_g1_i4 |
| 56 |  | TRINITY_DN7328_c0_g1_i4 |  | TRINITY_DN1587_c1_g1_i2 |
| 57 |  | TRINITY_DN3806_c0_g2_i3 |  | TRINITY_DN5865_c0_g1_i3 |
| 58 |  | TRINITY_DN5266_c0_g1_i11 |  | TRINITY_DN11397_c0_g1_i1 |
| 59 |  | TRINITY_DN32600_c0_g1_i1 |  | TRINITY_DN5919_c0_g1_i2 |
| 60 |  | TRINITY_DN37909_c0_g1_i1 |  | TRINITY_DN11901_c0_g2_i2 |
| 61 |  | TRINITY_DN13587_c0_g1_i1 |  | TRINITY_DN7492_c0_g1_i2 |
| 62 |  | TRINITY_DN27293_c0_g1_i1 |  | TRINITY_DN1983_c0_g1_i3 |
| 63 |  | TRINITY_DN10984_c0_g1_i1 |  | TRINITY_DN12080_c1_g1_i1 |
| 64 |  | TRINITY_DN7332_c0_g1_i8 |  | TRINITY_DN36167_c0_g1_i1 |
| 65 |  | TRINITY_DN30701_c0_g1_i1 |  | TRINITY_DN7528_c0_g1_i2 |
| 66 |  | TRINITY_DN17108_c0_g1_i1 |  |  |
| 67 |  | TRINITY_DN1779_c0_g3_i1 |  |  |
| 68 |  | TRINITY_DN19033_c0_g1_i4 |  |  |
| 69 |  | TRINITY_DN26777_c0_g1_i1 |  |  |
| 70 |  | TRINITY_DN23295_c0_g2_i4 |  |  |
| 71 |  | TRINITY_DN163_c1_g1_i1 |  |  |
| 72 |  | TRINITY_DN16002_c0_g1_i1 |  |  |
| 73 |  | TRINITY_DN64813_c0_g1_i1 |  |  |
| 74 |  | TRINITY_DN8988_c0_g2_i1 |  |  |
| 75 |  | TRINITY_DN57659_c0_g1_i1 |  |  |
| 76 |  | TRINITY_DN1894_c0_g1_i1 |  |  |

**Supplementary Table 5.** List of six selected genomes belonging to the Asteraceae family.

| Species | Subfamily | Accession | Assembly Size (bp) |
| --- | --- | --- | --- |
| *Artemisia annua* | Asteroideae | GCA_003112345.1 | 1,792,856,094 |
| *Cynara cardunculus* | Carduoideae | GCF_001531365.1 | 725,197,765 |
| *Erigeron canadensis* | Asteroideae | GCF_010389155.1 | 426,376,474 |
| *Helianthus annuus* | Asteroideae | GCF_002127325.2 | 3,010,047,587 |
| *Lactuca sativa* | Cichorioideae | GCF_002870075.3 | 2,388,971,937 |
| *Mikania micrantha* | Asteroideae | GCA_009363875.1 | 1,790,643,622 |

**Supplementary Table 6.** Top 8 of upregulated drought-responsive genes.

| No. | Transcript | log2FC | Matched query protein | Drought-responsive process |
| --- | --- | --- | --- | --- |
| 1 | TRINITY_DN22327_c0_g1_i1 | 1.65 | NP_564978.1 | ABA signaling |
|  |  |  | NP_563899.1 |  |
|  |  |  | AAN13027.1 |  |
|  |  |  | BAB08249.1 |  |
| 2 | TRINITY_DN1659_c0_g1_i3 | 1.68 | NP_564978.1 | ABA signaling |
|  |  |  | NP_563899.1 |  |
|  |  |  | AAN13027.1 |  |
|  |  |  | BAB08249.1 |  |
|  |  |  | BAA97215.1 |  |
| 3 | TRINITY_DN4970_c1_g1_i2 | 1.73 | NP_564978.1 | ABA signaling |
|  |  |  | NP_563899.1 |  |
|  |  |  | AAN13027.1 |  |
|  |  |  | BAB08249.1 |  |
|  |  |  | BAA97215.1 |  |
| 4 | TRINITY_DN14995_c0_g1_i1 | 1.77 | AED91326.1 | ABA signaling |
|  |  |  | AEE78672.1 |  |
|  |  |  | AED98274.1 |  |
|  |  |  | AEE33750.1 |  |
|  |  |  | NP_192695.1 | MAPK and Calcium signaling |
|  |  |  | NP_174807.1 |  |
|  |  |  | NP_197748.1 |  |
|  |  |  | NP_191312.2 |  |
| 5 | TRINITY_DN1678_c0_g1_i2 | 1.84 | NP_565030.1 | ABA signaling |
|  |  |  | Q9M9E1.1 |  |
|  |  |  | Q7PC88.1 |  |
|  |  |  | Q8GZ52.2 |  |
| 6 | TRINITY_DN17252_c0_g1_i1 | 1.92 | AED91326.1 | ABA signaling |
|  |  |  | AEE78672.1 |  |
|  |  |  | AED98274.1 |  |
|  |  |  | AEE33750.1 |  |
|  |  |  | NP_192695.1 | MAPK and Calcium signaling |
|  |  |  | NP_174807.1 |  |
|  |  |  | NP_197748.1 |  |
|  |  |  | NP_191312.2 |  |
| 7 | TRINITY_DN73142_c0_g1_i1 | 2.09 | NP_564978.1 | ABA signaling |
|  |  |  | AAN13027.1 |  |
|  |  |  | BAB08249.1 |  |
|  |  |  | BAA97215.1 |  |
| 8 | TRINITY_DN15780_c0_g3_i1 | 2.36 | NP_564978.1 | ABA signaling |
|  |  |  | NP_563899.1 |  |
|  |  |  | AAN13027.1 |  |
|  |  |  | BAB08249.1 |  |
